# RNA-Seq Based Transcriptome Analysis of *Aspergillus oryzae* DSM 1863 Grown on Glucose, Acetate and an Aqueous Condensate from the Fast Pyrolysis of Wheat Straw

**DOI:** 10.3390/jof8080765

**Published:** 2022-07-23

**Authors:** Christin Kubisch, Aline Kövilein, Habibu Aliyu, Katrin Ochsenreither

**Affiliations:** Institute of Process Engineering in Life Science 2: Technical Biology, Karlsruhe Institute of Technology, 76131 Karlsruhe, Germany; aline.koevilein@kit.edu (A.K.); habibu.aliyu@kit.edu (H.A.); katrin.ochsenreither@kit.edu (K.O.)

**Keywords:** filamentous fungi, acetate, pyrolytic aqueous condensate, RNA-seq analysis

## Abstract

Due to its acetate content, the pyrolytic aqueous condensate (PAC) formed during the fast pyrolysis of wheat straw could provide an inexpensive substrate for microbial fermentation. However, PAC also contains several inhibitors that make its detoxification inevitable. In our study, we examined the transcriptional response of *Aspergillus oryzae* to cultivation on 20% detoxified PAC, pure acetate and glucose using RNA-seq analysis. Functional enrichment analysis of 3463 significantly differentially expressed (log_2_FC >2 & FDR < 0.05) genes revealed similar metabolic tendencies for both acetate and PAC, as upregulated genes in these cultures were mainly associated with ribosomes and RNA processing, whereas transmembrane transport was downregulated. Unsurprisingly, metabolic pathway analysis revealed that glycolysis/gluconeogenesis and starch and sucrose metabolism were upregulated for glucose, whereas glyoxylate and the tricarboxylic acid (TCA) cycle were important carbon utilization pathways for acetate and PAC, respectively. Moreover, genes involved in the biosynthesis of various amino acids such as arginine, serine, cysteine and tryptophan showed higher expression in the acetate-containing cultures. Direct comparison of the transcriptome profiles of acetate and PAC revealed that pyruvate metabolism was the only significantly different metabolic pathway and was overexpressed in the PAC cultures. Upregulated genes included those for methylglyoxal degradation and alcohol dehydrogenases, which thus represent potential targets for the further improvement of fungal PAC tolerance.

## 1. Introduction

Lignocellulosic residues represent a globally abundant renewable resource with great potential to replace fossil-based processes for the production of value-added chemicals and fuels. The fast pyrolysis of wheat straw as performed in the bioliq^®^ plant at the Karlsruhe Institute of Technology (KIT) [1] may provide such an alternative to conventional fuel production. However, the pyrolytic aqueous condensate (PAC) formed during this process is hardly suitable for an energetic use due to its comparatively low heating value [2], and therefore currently accumulates as an unexploited side stream.

The organic carbons contained in the condensate render it a promising substrate for microbial fermentation with *Aspergillus oryzae*, as this filamentous fungus is capable of metabolizing the two major PAC components, acetate and acetol (Table A1, see Appendix A) [3,4]. *A. oryzae* is widely used in the traditional fermentative production of foods and beverages like soy sauce, miso paste and sake, but it also shows great potential for the secretion of hydrolytic enzymes [5,6,7] and organic acids [3,8,9]. The fungus is very robust to various environmental conditions, which is also reflected in its increased tolerance to liquid pyrolysis products compared to several other fungal species [10]. However, growth-inhibiting substances contained in the condensate, such as cyclic ketones, furans and phenols [10], prevent fungal growth on PAC concentrations above 1.25% [11]. The removal of these inhibitors is a common strategy to increase the PAC tolerance of the fungus [11], but since any treatment of the condensate involves additional laboratory effort and cost, the generation of more resistant production strains seems to be the preferable solution. This can either be accomplished by a rather time-consuming strain adaptation [12,13], undirected mutagenesis [14] or targeted genetic engineering [15,16]. Identifying potential targets for strain engineering requires comprehensive knowledge about the metabolic dynamics of the organism that is to be optimized. Transcriptome analysis is an appropriate tool for this purpose, as it provides insights into the regulation of metabolic gene expression. However, for *Aspergillus* species, most of the existing transcriptome studies related to lignocellulose focus on the identification of carbohydrate-active enzymes (CAZymes) [17,18,19,20] rather than the inhibitory effect of lignocellulose-degradation products.

This study therefore aims to provide insight into the transcriptional response of *A. oryzae* to cultivation in media containing PAC as the sole carbon source (C-source). Gene expression was compared with both pure acetate and glucose, the current standard substrate for most microbial fermentations. To allow the utilization of PAC as the sole C-source, pretreatment with a combination of overliming, rotary evaporation and activated carbon was required. By identifying potential targets for an improvement of fungal PAC tolerance, this study provides the basis for a potential reduction in pretreatment effort in the future.

## 2. Materials and Methods

### 2.1. Microorganism

*Aspergillus oryzae* DSM 1863 was obtained from the DSMZ German Collection of Microorganisms and Cell Cultures (Deutsche Sammlung von Mikroorganismen und Zellkulturen GmbH, Braunschweig, Germany). The storage of the fungus was carried out at −80 °C as aliquots of 50% glycerol conidia stock solution prepared according to [3].

### 2.2. Media and Cultivation Conditions

The medium for the *A. oryzae* cultivations was composed of 4 g/L (NH_4_)_2_SO_4_, 0.75 g/L KH_2_PO_4_, 0.98 g/L K_2_HPO_4_, 0.1 g/L MgSO_4_·7H_2_O, 0.1 g/L CaCl_2_·2H_2_O, 5 mg/L NaCl, 5 mg/L FeSO_4_·7H_2_O [9] and 2 mL/L Hutner’s Trace Element solution, with the latter containing 5 g/L FeSO_4_·7H_2_O, 50 g/L EDTA-Na_2_, 22 g/L ZnSO_4_·7H_2_O, 11 g/L H_3_BO_3_, 5 g/L MnCl_2_·4H_2_O, 1.6 g/L CoCl_2_·6H_2_O, 1.6 g/L CuSO_4_·5H_2_O and 1.1 g/L (NH_4_)_6_Mo_7_O_24_·4H_2_O, pH 6.5 [21].

Glucose, acetate and 20% detoxified PAC were used as carbon sources. The concentration of the first two substrates was chosen according to the amount of acetate present in the PAC to ensure comparability between the individual cultures. Glucose and acetate were added directly to the medium, and the pH of the acetate medium was adjusted to 6.5 using NaOH pellets. Both media were then autoclaved at 121 °C for 20 min. By contrast, the medium containing PAC was prepared without the C-source as a 2 × concentrated stock solution. After autoclaving, the medium was supplemented to 1 × concentration by adding sterile ultrapure water and 20% detoxified sterile-filtered PAC. Cultivation was performed in 500 mL shake flasks, each containing 100 mL of medium and 1 mL of a 10% Tween^®^ 80 solution to prevent the fungus from adhering to the flask walls. The flasks were inoculated by adding 1 mL of spore suspension (c = 3∙10^7^ spores/mL), and the cultures were incubated at 30 °C and 100 rpm for 48–72 h. In the preliminary experiment, a daily sampling of 1.9 mL was performed to follow substrate consumption during the cultivation, and the remaining content of the flask was used for biomass determination. All experiments were performed as biological triplicates.

### 2.3. Formation and Detoxification of the PAC

The PAC used in this work was formed in the second condensation step (25–30 °C) of the fast pyrolysis, which represents the first part of the bioliq^®^ process performed at KIT [1]. Prior to its utilization as substrate in fungal fermentation, the PAC was detoxified by a combination of overliming, rotary evaporation and a subsequent activated carbon treatment. For the overliming treatment, the pH of the PAC was increased to a value of 10 using solid Ca(OH)_2_. The condensate was then incubated in an oil bath at 80 °C for 4 h with continuous stirring. After the incubation, the condensate was centrifuged at 4700× *g* for 10 min to remove precipitates, and the supernatant was filtered using a paper filter (Macherey-Nagel, type MN615, cellulose). The pH of the filtrate was adjusted to 6.5 using 96% H_2_SO_4_ and the condensate was then further processed via rotary evaporation. For this treatment, the PAC was heated to 80 °C in an oil bath under constant rotation at 90 rpm. The pressure was gradually reduced to 40 mbar, and the PAC was refilled with ultrapure water to the initial volume to prevent the concentration of non-volatile toxins. Finally, 10% (*w*/*v*) activated carbon was added to the PAC, and the suspension was incubated for 1 h at room temperature with constant stirring. The carbon was then separated by centrifugation using the aforementioned parameters, and the supernatant was further purified by two successive vacuum-filtration steps to remove residual carbon particles. The first step, using a Büchner funnel and two layers of filter paper (Whatman, Buckinghamshire, UK, type 595), was followed by bottle top filtration (Nalgene Rapid-Flow, PES membrane, pore size 0.2 µm, Thermo Scientific, Waltham, MA, USA). The analysis of the PAC composition both before and after the treatment was performed by gas chromatography/mass spectrometry (GC/MS) at the Thünen Institute of Wood Research in Hamburg, Germany [22].

### 2.4. RNA Isolation, Library Preparation and Sequencing

To ensure comparable growth phases for all substrates tested, cell harvest for RNA isolation was performed at different time points of the cultivation. While sampling for glucose and pure acetate was performed after 24 h, the biomass of the PAC cultures was harvested after 48 h. For the biomass harvest, the content of the entire shake flask was poured through a Miracloth filter (Merck, Darmstadt, Germany) and then rinsed with ultrapure water. The washed biomass was then snap-frozen with liquid nitrogen and stored at −80 °C until further use. Total RNA isolation, the poly A enrichment of mRNA and the construction of Illumina-stranded TruSeq RNA libraries as well as mRNA sequencing (Illumina NextSeq, 75 bp, paired-end), were all performed by Microsynth (Balgach, Switzerland).

### 2.5. RNA-Seq Data Analysis

Quality control of the raw reads was performed with fastQC v0.11.9 [23] and fastp v0.20.1 using the default settings, except the cut window size (−W), the minimum length (−L) and the qualified quality Phred (−q) set at 16, 50 and 30, respectively. We mapped the high-quality reads against the *A. oryzae* RIB40 genome [24] using STAR v2.7.10a [25] and generated read counts with RSEM v1.2.28 [26] under default settings. The gene count data were filtered (minimal counts per million (CPM) = 0.5 in at least one library) and transformed based on the regularized logarithm transformation (rlog) method implemented in iDEP.95 [27]. iDEP.95 was also used to perform and visualize the principal component analysis (PCA) of the transformed read data as well as for differential gene expression (DGE) analysis using the DESeq2 package [28]. For the DGE analysis, the log_2_ fold change (log_2_FC) and false discovery rate (FDR) thresholds were set at >2 and <0.05, respectively. To gain insights into the functional implication of the observed differential gene expression, we performed GO (gene ontology) term enrichment analysis in iDEP.95 and KEGG pathway analysis using Pathview Web [29]. The additional annotation of *A. oryzae* RIB40 predicted proteins was performed with eggNOG mapper v2.1.7 [30] and uniprot [31].

### 2.6. Analytics

#### 2.6.1. Quantification of the Fungal Cell Dry Weight (CDW)

Fungal dry cell weight was determined by filtering the content of an entire shake flask through a pre-weighed paper filter. The retained biomass was thoroughly washed with ultrapure water, and the filters were dried in an oven at 70 °C until no more change in weight was detected. The dried filters were weighed with a precision scale, and the CDW was expressed in g/L after subtracting the blank weight of the filters.

#### 2.6.2. Enzymatic Acetate Assay

The concentration of acetate in the PAC cultures was quantified using an enzyme assay (10148261035, R-Biopharm AG, Darmstadt, Germany). The measurements were performed according to the manufacturer’s instructions, except that all volumes were reduced to one quarter.

#### 2.6.3. HPLC Analysis

The concentrations of glucose and acetate in the cultures without PAC were determined by reverse-phase HPLC (Agilent 1100 Series, Agilent, Waldbronn, Germany) using a Rezex ROA organic acid H+ (8%) column (300 × 7.8 mm, 8 µm particle size; Phenomenex) and a Rezex ROA organic acid H+ (8%) guard column (50 × 7.8 mm). For the quantification of the glucose content, the oven temperature was set at 50 °C, and 5 mM H_2_SO_4_ was used as eluent, while 60 °C and 3 mM H_2_SO_4_ were chosen for the determination of the acetate concentration. In both cases, elution was performed isocratically at a flow rate of 0.5 mL/min. Glucose was detected using a refractive index detector, while a UV detector with a wavelength of 220 nm was used for the organic acid. The actual quantification was performed via calibration curves in the range of 0.1–5 g/L.

## 3. Results and Discussion

### 3.1. Characterization of the Exponential Growth Phase in A. oryzae Shake Flask Cultures

**Figure 1:**
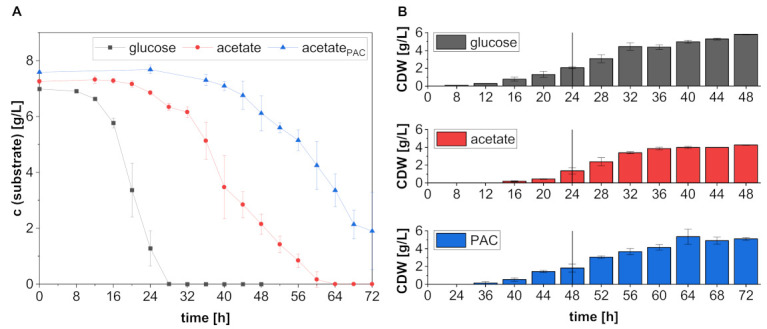
Substrate consumption (**A**) and biomass formation (**B**) in *A. oryzae* cultures containing glucose, acetate and 20% detoxified pyrolytic aqueous condensate (PAC). The vertical lines indicate the time of sampling for RNA isolation. The data are means of three biological replicates, and the error bars represent the standard deviation.

To ensure the construction of comparable RNA libraries for all substrates, the growth of *A. oryzae* on the different C-sources was first characterized in a preliminary shake flask experiment. As shown in Figure 1, substrate concentrations at the beginning of cultivation ranged from 6.99 ± 0.03 g/L to 7.48 ± 0.19 g/L.

Besides these minor differences in the initial substrate concentration, the cultures differed primarily in the duration of their lag phase. While, in the cultures with glucose, the substrate conversion and biomass formation already started after 8–12 h, the lag phase in the two other cultures was considerably prolonged. For the flasks containing pure acetate, a formation of 0.19 ± 0.06 g/L biomass was observed after 16 h, although no decrease in substrate concentration could be detected. This indicates that, at that time, the fungus was still consuming the reserves stored in the conidia as well as the remaining glycerol from the spore suspension. The fungal ability to metabolize polyol has been reported by Ochsenreither et al. [9]. However, after these first 16 h, the acetate concentration began to decrease gradually. The longest lag phase was observed in the PAC cultures, as in these flasks, an onset of acetate consumption and biomass formation became detectable only after 36 h of cultivation. This is most likely due to the presence of growth-inhibitory components in the pyrolysis condensate. According to Dörsam et al., among the compounds that were still present in the PAC after the detoxification procedure (Table A1), hydroxyacetone (acetol), 2-cyclopenten-1-one and furfural were the most relevant growth inhibitors, whereas ethylene glycol and γ-butyrolactone were found to be rather harmless to *A. oryzae* [10]. Considering that we used 20% PAC, only the concentration of 2-cyclopenten-1-one was still close to the growth limit of 0.00625 wt.% determined by Dörsam et al. [10]. However, it can be assumed that some inhibition already occurs at concentrations below the growth limit.

By the time growth and acetate metabolization were just starting in the flasks with PAC, the substrate had already been completely consumed in the glucose-containing cultures. However, the depletion of glucose did not result in transition to the stationary phase. Rather, after a short stagnation between 32–36 h, the biomass increased again and resulted in a final cell dry weight (CDW) of 5.80 ± 0.04 g/L. Apparently, after glucose depletion, the fungus utilized metabolic intermediates, such as organic acids, for growth. In the acetate cultures, the end of the exponential growth phase was reached after 36 h, and the biomass concentration increased only slightly from 3.87 ± 0.14 g/L to 4.26 ± 0.04 g/L during the last 12 h of cultivation. As Figure 1 indicates, this retardation in cell growth cannot be attributed to substrate depletion, since, at this time, 5.13 ± 0.66 g/L of acetate was still present in the medium. Furthermore, as there was also no reduction in acetate consumption, it can be assumed that the fungus was still metabolically active. This activity resulted in the complete depletion of the substrate after 64 h. However, at this stage of cultivation, no further determination of CDW was performed, since the main objective of the experiment was to characterize the exponential growth phase.

There was hardly any exponential phase observed during growth on PAC, as the increase in biomass in these cultures was rather linear. The maximum CDW of 5.35 ± 0.86 g/L was reached after 64 h of cultivation and biomass formation largely stagnated afterwards. As already observed for the acetate cultures, no reduction in substrate consumption was detected. However, due to the later onset of acetate metabolization in the PAC cultures, 1.26 ± 0.38 g/L substrate remained unused at the end of the experiment.

Interestingly, the maximum CDW achieved on PAC was only slightly lower than in the glucose cultures, while the lowest biomass concentration was obtained for pure acetate. This suggests that the fungus is able to utilize other PAC components in addition to acetate. Moreover, the fact that the fungus was able to grow normally on PAC after overcoming the initial lag phase may indicate that the inhibitors either exerted their negative influence mainly on the early developmental stages of the fungus or that the toxic substances can at least be partially degraded. For example, it has been demonstrated for some *Aspergillus* strains that they are capable of converting furanic compounds like HMF or furfural [32,33].

In summary, this experiment demonstrated the necessity to first characterize growth on the different C-sources to ensure similar cultivation conditions by the time of RNA sampling. This is important because He et al. revealed that the growth phase has a major impact on gene expression in *A. oryzae*, with the greatest differences occurring during the transition from the adaptive to the exponential phase [34]. As indicated by the vertical line in Figure 1, we therefore performed the RNA sampling after 24 h in the flasks containing glucose and acetate, whereas the biomass of the PAC cultures was harvested after 48 h.

### 3.2. General Characteristics of the RNA-Seq Data

After determination of the ideal sampling time, the actual RNA isolation and sequencing was performed. We obtained 184.30 × 2 million reads (mean read length: 75 bp), ranging between 18.72 and 22.87 million reads per sample (Table 1).

**Figure 2:**
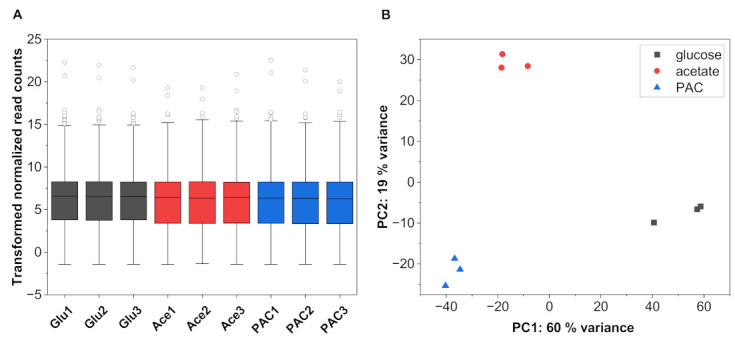
Box plot of the transformed and normalized read count data (**A**) and principal component analysis (PCA) showing the variance between the individual samples (**B**).

After removing the adaptor sequences as well as low-quality and ambiguous reads, the final RNA-seq data comprised 14.20–17.90 million clean reads per sample. These clean reads were aligned against the *A. oryzae* RIB 40 genome (ASM18445v3) [24] and could be uniquely mapped at an average rate of 94%, confirming the high quality of our RNA-seq data. Only PAC sample 3 showed a slightly lower but still acceptable alignment ratio of 86%. Overall, 9634 genes passed the set filter of 0.5 CPM in a minimum of one library and formed the basis of subsequent analyses. Figure 2 displays the distribution and the principal component analysis (PCA) of the transformed and normalized gene counts.

The PCA showed a close clustering of the individual samples within a triplicate and a clear separation between the different C-sources, suggesting high repeatability of the RNA-seq data. Approximately 79% of the transcriptome variation among the compared samples could be explained in PC1 (60%) and PC2 (19%), indicating clear variation in transcriptome profiles among the three treatment regimes.

### 3.3. Differential Gene Expression during Growth on the Different C-Sources

**Figure 3:**
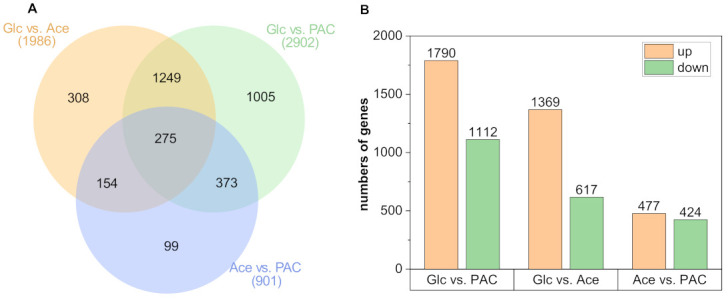
Differentially expressed genes of *A. oryzae* during cultivation with 20% detoxified PAC, acetate or glucose. Venn diagram showing the distribution and overlap of DGE for the comparisons of the different substrates (**A**). Number of up- and downregulated genes for the individual comparisons (**B**).

DGE analysis revealed 3463 significantly differentially expressed (FDR ≤ 0.05 and log_2_FC ≥ 2) genes, of which only 275 genes (7.94%) showed altered expression in all three conditions (Figure 3A). In comparison to glucose, growth on PAC and acetate led to significant expression changes for 2902 and 1986 genes, respectively. By contrast, when comparing acetate and PAC, only 901 genes showed significant differential expression. This value is considerably lower than for the other conditions, which is probably due to the fact that acetate is one of the main components of PAC (Table A1). Therefore, many of the changes in transcription induced by acetate are probably also found during growth on PAC. Conversely, this may also explain why the number of differentially expressed genes for the comparison between glucose and PAC was 32% higher than for glucose and pure acetate, as it is very likely that the additional PAC components cause changes in fungal gene expression that are beyond those induced by acetate. 

Among the 2902 genes that were differentially expressed during growth on glucose and PAC, there was a set of 1790 genes being upregulated, whereas 1112 genes were downregulated (Figure 3B). However, even more remarkable when comparing these two C-sources was the relatively large number of genes (1005) that were uniquely differentially expressed between these two conditions. This corresponds to 29.02% of the 3463 genes for which a significant change in expression was detected during this RNA-seq experiment. By contrast, of the 1369 and 617 genes that were upregulated and downregulated, respectively, when glucose and pure acetate were compared, only 308 were found to be differentially expressed exclusively between these two C-sources. Instead, a majority of 1294 genes showed an identical transcriptional regulation as observed between glucose and PAC. For the reason already stated above, the lowest number of only 477 upregulated and 424 downregulated genes was found for the comparison of acetate and PAC, of which only 99 were differentially expressed solely between these two C-sources. However, these genes are of particular interest for understanding what changes in transcriptional regulation are triggered by the other components of PAC rather than acetate.

### 3.4. Functional Enrichment and Pathway Analyses 

**Figure A1:**
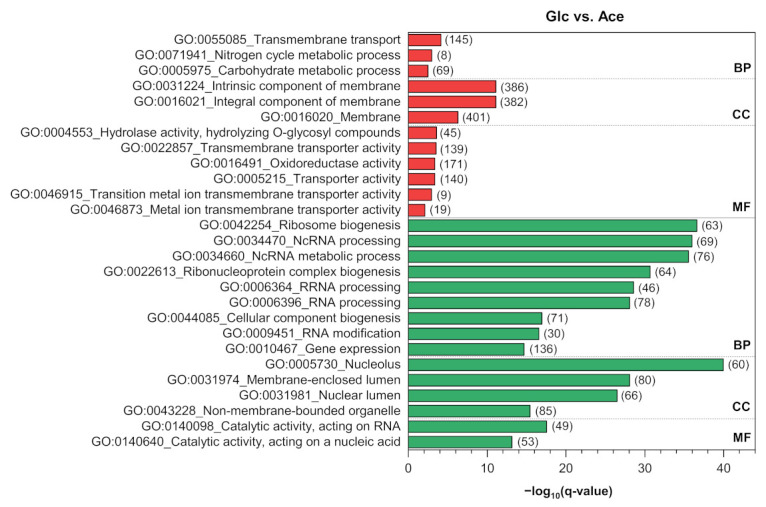
Functional enrichment analysis of genes being differentially expressed during cultivation of *A. oryzae* on glucose (Glc) compared to pure acetate (Ace). BP = biological process, CC = cellular component and MF = molecular function.

**Figure A2:**
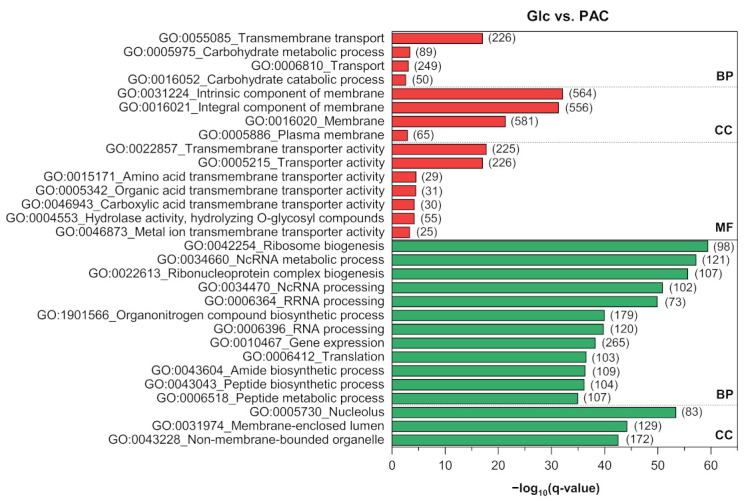
Functional enrichment analysis of genes being differentially expressed during the cultivation of *A. oryzae* on glucose (Glc) compared to the pyrolytic aqueous condensate (PAC). BP = biological process, CC = cellular component and MF = molecular function.

To gain insight into the functional categories associated with the differentially expressed genes, we performed a gene ontology (GO) enrichment analysis. Based on this analysis, upregulated genes in the glucose control compared with both pure acetate and PAC were primarily enriched in membrane (GO:0016020)-related activities and transmembrane transport (Figure A1 and Figure A2).

Unsurprisingly, several of the genes assigned to the latter GO term (GO:0055085) were found to be putative sugar transporters of the major facilitator superfamily (MFS) (Supplementary Table S1). The MFS is one of the largest transporter families in living organisms, whose members can transport a broad spectrum of substances. For instance, in addition to sugar transporters, MFS also includes the families of proton-dependent oligopeptide transporters (POT) and monocarboxylate transporters (MCT) [35], for which we also found some upregulated genes in our data sets. It was rather unexpected that the latter were upregulated in the glucose cultures and not during growth on acetate or PAC, since a gene encoding an MCT in *Aspergillus* species was described as showing increased expression when acetate was used as the sole C-source [36]. However, in this study it has also been suggested that the transporter plays a role in the drug resistance of the fungus. Consistent with these findings, several genes belonging to the ATP-binding cassette (ABC) transporters superfamily and thus associated with multidrug resistance (MDR) were found to be upregulated in our glucose cultures. Initially, this also seemed contrary to our expectations, since the involvement of an ABC transporter in acetate tolerance had been reported in *S. cerevisiae* [37]. Moreover, there was another study showing that the growth of the same organism on a lignocellulosic hydrosylate led to the increased expression of MDR transporters [38].

However, it has been hypothesized that ABC and MFS transporters may also be associated with the synthesis and secretion of secondary metabolites (SM) and mycotoxins [39,40]. We therefore assume that the export of SM might be impaired in the acetate-containing cultures, leading to the higher relative expression of transporters in the glucose controls. For example, we identified the *wykF* gene to be upregulated when glucose and PAC were compared. The gene encodes an H+/oligopeptide symporter of the POT family involved in the secretion of wyk-1. This compound was described as a dipeptidyl peptidase IV inhibitor produced by the *A. oryzae* strain AO-1 [41]. In addition, the MFS transporter genes *cpaT* (AO090026000005), *hepF* (AO090011000413) *kojT* (AO090113000138) and a gene of the MCT family (AO090003001541) also showed increased expression in the glucose cultures. These genes are responsible for the export of the antimicrobial agents cyclopiazonic acid [42], heptelidic acid [43], kojic acid [44] and aspergillic acid [45]. A closer look at the associated gene clusters reveals that not only the transport of these secondary metabolites but also their synthesis seems to be partially downregulated in the acetate-containing cultures (see Appendix B for further discussion). However, since, even among the currently uncharacterized transporters in our data sets, most genes belong to the MFS or at least contain a corresponding domain, it seems conceivable that there are even more of these genes involved in SM secretion. The genes that did not belong to the MFS were primarily ion transporters for iron, calcium, potassium and sodium, but also ammonia and amino acid permeases. For the latter, it has been described in both bacteria and yeasts that they can be inhibited by weak organic acids such as acetic acid [46,47], which could thus explain the downregulation of these genes in the acetate-containing cultures.

**Figure A3:**
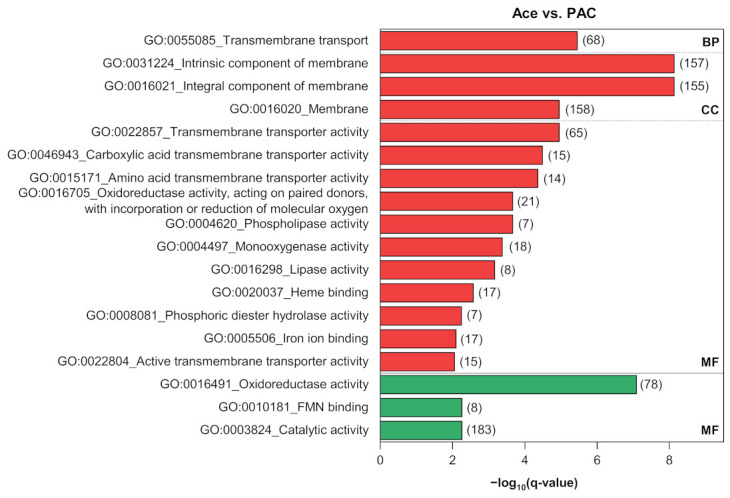
Functional enrichment analysis of genes being differentially expressed during the cultivation of *A. oryzae* on pure acetate (Ace) compared to the pyrolytic aqueous condensate (PAC). BP = biological process, CC = cellular component and MF = molecular function.

The fact that transmembrane transport also played a central role in the direct comparison of pure acetate and PAC, and that this GO term was downregulated during growth on the latter (Figure A3) suggests an additional inhibitory effect of the other PAC components on cellular transport processes. One example is the inhibition of amino acid transport by furfural, as demonstrated in *S. cerevisiae* [48]. Consistent with this study, ~20% of the 68 genes associated with transmembrane transport were identified as amino acid transporters (Supplementary Table S1).

Despite these additional PAC components, the GO terms that were found to be upregulated in comparison to glucose were very similar for both acetate-containing cultures and were mainly related to ribosomes and non-coding RNA processing (Figure A1 and Figure A2). It can therefore be assumed that the upregulation is primarily caused by acetate or the high salt concentration in these cultures resulting from the pH adjustment. This was consistent with a transcriptional analysis on the salt-tolerance mechanism of the yeast *Zygosaccharomyces rouxii*, which appears to involve the increased expression of genes associated with ribosome synthesis and RNA processing [49]. Similar results were obtained in a transcriptome analysis investigating the effects of a sugarcane biomass hydrolysate and its main inhibitors on *Pichia pastoris*. Among the GO terms that were overexpressed in response to hydrolysate addition were primarily those related to processing of noncoding RNA, and, based on the analysis of the individual inhibitors, this observation could be attributed to acetate [50].

Since our functional enrichment analysis also included GO terms assigned to the carbohydrate-degradation process (GO:0016052) and the carbohydrate-metabolic process (GO:0005975), we performed pathway analysis based on the Kyoto Encyclopedia of Genes and Genomes (KEGG) database to further assess the function of the differentially expressed genes in the fungal metabolism. The analysis revealed that 18 metabolic pathways were differentially expressed between glucose and acetate, while 33 pathways showed an altered expression when the cultures containing glucose and PAC were compared (Table 2). This once again indicates that the additional components of the pyrolysis condensate exert an impact on the transcriptional regulation of the fungus. 

Most of the differentially expressed genes for both comparisons were assigned to metabolic pathways and the synthesis of secondary metabolites. However, since “metabolic pathways” can be considered a generic term encompassing a whole range of cellular processes, we also focused on subordinate pathways that showed significant differences in expression. Among these, the biosynthesis of amino acids and carbon metabolism showed the largest set size, regardless of whether glucose was compared with PAC or pure acetate. In the following sections, we will therefore take a closer look at these two metabolic pathways and also highlight some relevant secondary metabolite clusters (Appendix B). A comparison of the transcriptome during growth on acetate and PAC revealed that pyruvate metabolism was the only significantly different metabolic pathway. Therefore, it is also described in more detail in the following section.

#### 3.4.1. Carbon Metabolism

As expected, the cultivation of *A. oryzae* on different substrates had a significant impact on the transcriptional regulation of fungal carbon metabolism. During growth on acetate and PAC, “2-oxocarboxylic acid metabolism” and the “glyoxylate and dicarboxylate metabolism” were found to be the most significantly affected metabolic pathways of carbon utilization (Table 2). In addition, the upregulation of the TCA cycle and closely associated oxidative phosphorylation occurred on PAC. Furthermore, it is not particularly surprising that “glycolysis/gluconeogenesis” and “starch and sucrose metabolism” were among the pathways that were upregulated on glucose, since these are central metabolic routes for the utilization of the monosaccharide and its polymers.

##### Starch and Sucrose Metabolism

Most of the genes involved in “starch and sucrose metabolism” were already included in the GO term “hydrolase activity, hydrolyzing O-glycosyl compounds” and code for a variety of oligosaccharide-degrading enzymes (Table 3). There was a general upregulation of this pathway when glucose was compared with both acetate and PAC, although two genes showed a decreased expression in the latter comparison. These involved a glucan-1,3-β-glucosidase (AO090038000279) and a beta-glucosidase gene (AO090701000841). The induction of fungal beta-glucosidases by lignocellulose-based hydrolysates has already been shown in literature [51]. But in contrast to PAC, these hydrolysates contained sugars as degradation products of cellulose and hemicellulose, which might have induced the expression of the enzymes. 

However, the majority of genes were upregulated in the glucose cultures, although it is well known that the expression of carbohydrate-degrading enzymes is usually suppressed via catabolite repression as long as glucose is present [52]. This regulatory system was extensively studied in *A. oryzae*, especially for starch-degrading enzymes such as alpha-amylase, for which we also observed high fold changes (Table 3). It was shown that the system allows some basal enzymatic activity despite the presence of glucose [53], whereas no activity was detected when acetate was used as the sole substrate [54]. These results support our observations of a lower relative expression of these genes in the acetate and PAC cultures.

In addition to enzymes catalyzing oligosaccharide degradation, the differentially expressed genes of this pathway also encoded several anabolic enzymes (Table 3). Among these were two enzymes involved in the formation of trehalose, a disaccharide consisting of two glucose units linked by an α,α-1,1-glycosidic bond. Trehalose plays a crucial role as a storage component in spores and during vegetative growth but has also been reported to act as a protectant against a variety of stressors such as heat or oxidative and osmotic stress [55,56] by stabilizing membranes [57] and proteins [58]. Therefore, we would have expected the upregulation of these genes in acetate-containing cultures. However, in addition to these functions, an involvement of the disaccharide and its biosynthetic genes in the regulation of glycolytic flux [59] and cell wall integrity [60] in filamentous fungi have previously been reported. The latter also matches the observed upregulation of “amino sugar and nucleotide sugar metabolism” in the glucose cultures, as this metabolic pathway involves genes associated with the synthesis and degradation of chitin, the major component of the fungal cell wall. Thammahong et al. studied the regulatory subunits of trehalose synthesis, *tslA* and *tslB*, in *A. fumigatus* and showed that the deletion of these genes resulted in increased sensitivity to cell-wall-perturbing agents. Moreover, TslA in particular interacts with the chitin synthase CsmA and affects its activity and cellular localization [61]. In accordance with these findings, we found the increased expression of an *A. oryzae* homolog to *tslB* (AO090005001531, 73% amino acid identity; Table 3), as well as the *csmA* gene (AO090026000321) in the glucose cultures. Furthermore, the upregulation of five other genes encoding chitin synthases was observed (Supplementary Table S2). However, this is in contrast to a study showing that the osmotic stress induced by high sodium acetate concentrations results in the increased expression of the chitin synthases *chsA-C* in *A. nidulans* [62]. 

In addition to the cell wall, the cellular membrane is also an important barrier against external stressors, with its composition varying greatly depending on environmental conditions. For example, in *S. cerevisiae*, the increased incorporation of oleic acid (C_18:1_) into the cell membrane enables the organism to overcome the toxic effects of ethanol [63]. Moreover, the heterologous expression of oleate Δ12 desaturases in the same organism improved its NaCl tolerance by increasing the fluidity of the membrane [64]. Accordingly, we observed the increased expression of two such ∆12-desaturase genes in our acetate-containing cultures, with one being exclusively upregulated in acetate (AO090001000224: log_2_FC = 1.04) and the other showing an increased expression for both C-sources (AO090010000714; log_2_FC_Acetate_ = 3.07 & log_2_FC_PAC_ = 3.12). These enzymes are required for the production of linoleic acid, a diunsaturated fatty acid (C_18:2_) whose intracellular content has been described as significantly increased in *A. oryzae* under high-salinity conditions [65].

##### Glycolysis, Glyoxylate and the TCA Cycle

**Figure 4:**
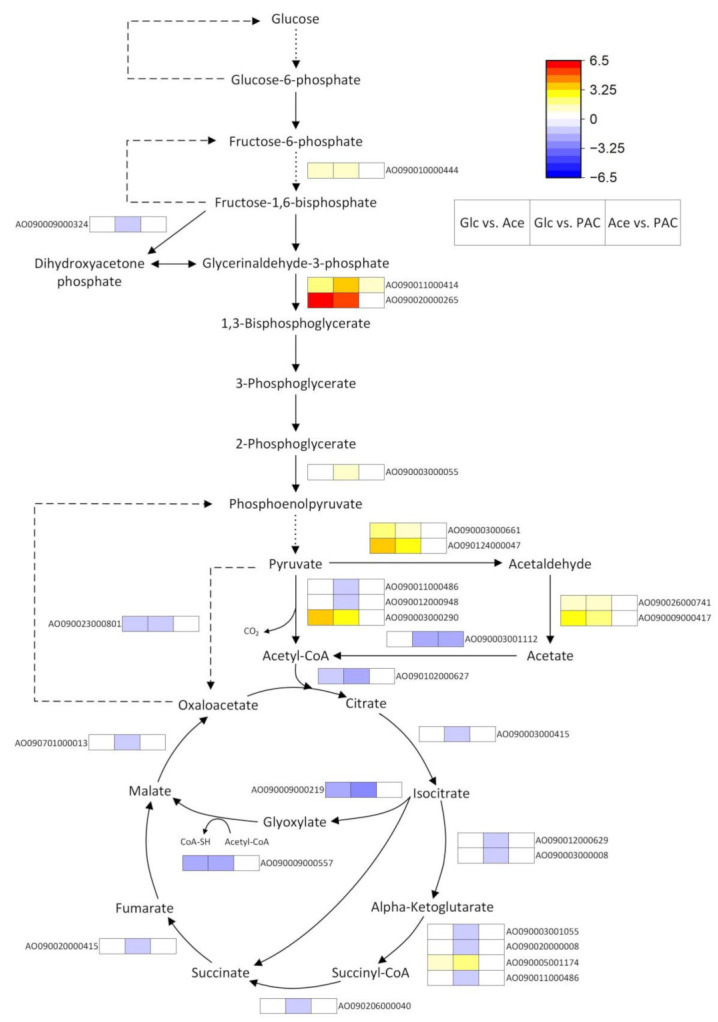
Genes of glycolysis, gluconeogenesis and the TCA cycle that were differentially expressed during the cultivation of *A. oryzae* on glucose (Glc), acetate (Ace) and PAC. The dashed and dotted lines indicate gluconeogenesis- and glycolysis-specific genes, respectively.

Contrary to our expectations, comparing the gene expression of glycolytic enzymes between glucose and the acetate-containing cultures revealed only a few differentially expressed genes (Figure 4). One possible reason for this observation could be that most glycolytic enzymes also catalyze the reverse reactions required for gluconeogenesis. Therefore, increased flux through this pathway in the cultures with acetate could have resulted in the low number of differences. However, since some of the reactions are not reversible and are thus specific to glycolysis or gluconeogenesis, at least the genes encoding these enzymes should be differentially expressed. Indeed, our data showed a slight downregulation of a gene that codes for the gluconeogenesis enzyme pyruvate carboxylase in the glucose cultures, whereas gene expression of the glycolysis-specific 6-phosphofructokinase (*pfk*) was increased. However, the *pfk* gene that was found to be upregulated (AO090010000444) encodes for *pfkB* and not *pfkA*. In *A. oryzae*, the expression of both *pfk* orthologues during growth on glucose has already been studied, and it was reported that *pfkB*, unlike *pfkA*, is even slightly repressed by glucose [66], suggesting that it is not the primary enzyme of glycolysis.

Glyceraldehyde 3-phosphate dehydrogenase (gpd) is also encoded by multiple genes in *Aspergillus* species, with *gpdA* certainly being involved in glycolysis [66]. However, similar to *pfk*, we found that primarily the other two *gpd* genes (*gpdB* and *gpdC*) were upregulated on glucose when compared with the acetate-containing cultures. The highest fold change was found for *gpdC* (AO090020000265), a gene whose exact function is unknown and which has a low similarity to *gpdA* (only 44% amino acid identity). In contrast, *gpdB* (AO090011000414) is known to be a part of the heptelidic acid gene cluster and is therefore discussed in more detail in the secondary metabolite section (Appendix B).

Of central importance in linking glycolysis and the TCA cycle is the pyruvate dehydrogenase multi-enzyme complex. It catalyzes the conversion of pyruvate to acetyl-CoA with the release of CO_2_ and is composed of three individual catalytic enzymes (E1–E3). Our data contained two differentially expressed genes encoding for the α subunit of E1, as well as the dihydrolipoamide dehydrogenase gene (E3). However, their regulation was quite ambiguous, because one of the E1 genes (AO090003000290) was highly upregulated on glucose, whereas the other (AO090012000948) as well as the E3 gene (AO090011000486) were slightly downregulated when the monosaccharide and PAC were compared (Figure 4). But since E3 is also a part of the alpha-ketoglutarate dehydrogenase complex, a key enzyme in the TCA cycle, its downregulation cannot be clearly attributed to glycolysis.

Pyruvate decarboxylase is another enzyme that catalyzes the cleavage of CO_2_ from pyruvate and for which we found two genes (AO090124000047 and AO090003000661) to be upregulated in the glucose cultures. The acetaldehyde formed in this reaction can be further converted either to ethanol by alcohol dehydrogenases (ADH) or to acetate by the action of aldehyde dehydrogenases. Whereas transcriptional regulation of ADHs was dependent on whether the enzyme was NADH- or NADPH-preferring (discussed in more detail in the pyruvate-metabolism section), the latter showed clear upregulation during growth on glucose. However, this upregulation implies underrepresentation in the other two cultures. Since these contain acetate as a substrate, which can directly enter the TCA cycle after a reaction with coenzyme A (CoA), the repression of genes coding for acetate-forming enzymes seems plausible. Enhanced chemical reaction with CoA in these cultures is also suggested by the upregulation of the acetyl-CoA synthetase gene, although it is unclear why higher expression occurred only for PAC and not for pure acetate.

Consistent with these findings, genes encoding carnitine O-acetyltransferases were also found to be upregulated only on PAC (AO090001000295, AO090026000404; Supplementary Table S2). Since the outer mitochondrial membrane is impermeable to acetyl-CoA, these enzymes are required to convert the acetyl-CoA formed by the cytosolic acetyl-CoA synthetase to O-acetylcarnitine, thus enabling entrance into the mitochondrion and the TCA cycle [67]. However, as previously described and also confirmed by the strong upregulation of the isocitrate lyase and malate synthase genes in our data, acetate is metabolized mainly via the glyoxylate cycle [68]. Since this process does not take place in the mitochondrion, our results regarding the carnithine-mediated acetate transport seem plausible. The glyoxylate cycle genes were also upregulated during growth on the pyrolysis condensate, but, in addition, the PAC cultures also showed an increased expression of almost the entire TCA cycle. The fact that TCA cycle genes were exclusively upregulated during growth in PAC indicates that this is a transcriptional response caused by the additional components of the condensate. This assumption is confirmed, for example, by a transcriptome analysis of *Kluyveromyces marxianus*, which also revealed the upregulation of most TCA genes during the growth of this yeast on an inhibitor mixture containing acetic acid, phenols, furfural and HMF [69]. Moreover, a metabolic flux analysis in glucose-limited *S. cerevisiae* chemostats showed that feeding 2.25 g/L furfural increased the specific rates of TCA and respiration by 50% each, thus also providing an explanation for the observed upregulation of oxidative phosphorylation in the PAC cultures (Table 2) [70].

#### 3.4.2. Biosynthesis of Amino Acids

As can be seen from Table 2, amino acids synthesis seems to be a key target in the regulation of the fungal gene expression during growth on the different C-sources. The pathview analysis revealed a significant downregulation of this pathway when glucose was compared to both acetate and PAC. Looking more closely at the individual amino acids, it becomes apparent that the “cysteine and methionine metabolism” showed the highest significance for both comparisons. The “tryptophan metabolism” was also significantly downregulated. Moreover, it contained the highest number of genes among all differentially expressed amino acid pathways with a set size of 16 and 20 genes for acetate and PAC, respectively. Besides these highly significant pathways, there were two more differentially expressed pathways, “glycine, serine and threonine metabolism” and “arginine and proline metabolism”, which could not be considered significant due to their p-value being greater than 0.05 (Table A2). Nevertheless, these pathways were chosen to be included in the analysis, as they might still contain genes relevant for the evaluation of the overall transcriptional regulation. 

##### Arginine and Proline Metabolism

Although the overall expression of the “arginine and proline metabolism” was not significantly altered in any of the tested conditions, the arginine-synthesis part of the pathway nevertheless showed comparable transcriptional regulation to that previously described by He et al. for high-salinity stress in *A. oryzae* [65]. In their work, salt concentrations of 5–15 g/L NaCl induced an upregulation of almost every gene involved in the formation of arginine from the citrate cycle intermediate alpha-ketoglutarate.

Due to the pH adjustment of our medium containing pure acetate using NaOH, a high sodium-ion concentration was present in these cultures. However, as indicated in the Supplementary Table S2, even more genes of this pathway were upregulated on PAC and also the fold changes were slightly higher than when acetate was used as substrate. This can most likely be attributed to the increased salt-ion concentration resulting from the overliming treatment of the pyrolysis condensate with Ca(OH)_2_. The upregulation of genes involved in arginine synthesis also being observed in the presence of calcium ions suggests that the transcriptional response may be triggered not only by NaCl but also by other osmotic stressors. This assumption is supported by the work of Ma et al. who reported the intracellular accumulation of free arginine in the cytosol of *A. oryzae* in response to ethanol stress [71]. Moreover, in the yeast *Candida glabrata*, not only an upregulation of the arginine synthesis was observed but also the downregulation of the genes involved in the degradation of this amino acid [72]. This is partially consistent with our results, as we observed the decreased expression of the arginase gene (AO090003000697) during growth on acetate compared to the glucose control (log_2_FC = −1.18, Supplementary Table S2). The positive influence of this amino acid on the integrity of the cell wall and membrane [73], as well as its activity as a suppressor of protein aggregation [74], has already been described in the literature and may provide a possible explanation for the increased arginine demand of cells during osmotic stress.

##### Glycine, Serine and Threonine Metabolism

“Glycine, serine and threonine metabolism” is another pathway whose expression was not significantly changed but which nevertheless should not be neglected due to its close association to other relevant amino acid pathways like tryptophan and cysteine metabolism.

An interesting gene of this pathway, which was upregulated in PAC relative to glucose (log_2_FC = 1.09), encodes the betaine-aldehyde dehydrogenase (AO090103000021). This enzyme catalyzes the second step of the synthesis of glycine-betaine, a trimethylated derivative of the amino acid glycine, which has been described as an osmoregulator, especially in plants [75] and bacteria [76]. Accordingly, the increased expression of the betaine-aldehyde dehydrogenase gene in the PAC-containing cultures could possibly be a reaction to the high osmolarity of the medium. However, it is still unclear whether glycine-betaine also contributes to the regulation of osmotic stress in *Aspergillus* species. Whereas Kelavkar et al. reported that the external addition of this amino acid derivative led to an improved growth of *A. repens* under high NaCl conditions [77], Lambou et al. suggested glycine-betaine biosynthesis to be a salvage pathway for the utilization of unusual nitrogen or carbon sources under nutrient starvation conditions [78].

**Figure 5:**
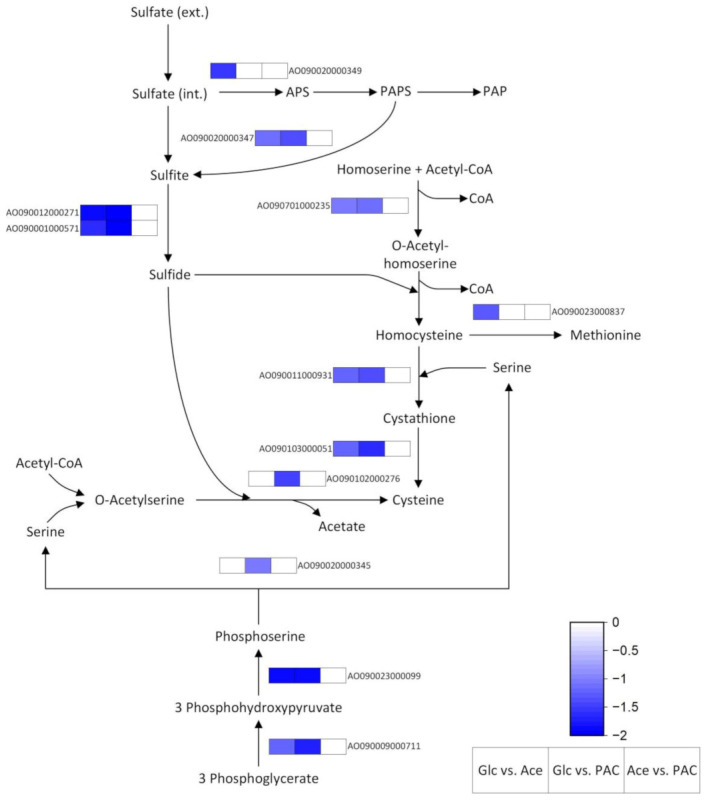
Differentially expressed genes of cysteine and methionine metabolism, as well as the closely related serine synthesis and sulfur metabolism, during the cultivation of *A. oryzae* on glucose (Glc), acetate (Ace) and the pyrolytic aqueous condensate (PAC). APS = adenosine 5′-phosphosulfate; PAPS = 3′-phosphoadenosin-5′-phosphosulfat; PAP = 3-phosphoadenosine 5-phosphate.

In addition to this glycine-related gene, DGE on acetate and PAC mainly involved genes for the synthesis of serine and its conversion to other amino acids and metabolites (Supplementary Table S2). Serine formation occurs in three enzymatic steps with the glycolysis intermediate 3-phosphoglycerate serving as the starting substance (Figure 5).

In comparison to glucose, this pathway was identified to be entirely upregulated when the fungus was grown on PAC, whereas, on pure acetate, the gene for the final reaction step was excluded by our strict filter despite a slight upregulation (log_2_FC = 0.83). The increased expression of this pathway might be related to the higher flux through the glyoxylate cycle during growth on acetate-containing media. For example, a metabolic flux analysis using *A. niger* previously revealed that the overexpression of isocitrate lyase, the key enzyme of the glyoxylate shunt, resulted in increased flux towards serine [79].

##### Cysteine and Its Role in Antioxidant Defense

The upregulation of genes involved in serine formation could also be explained by an increased demand for amino acids such as tryptophan or cysteine, which require serine for their synthesis. Indeed, both acetate-containing cultures showed the increased expression of almost the entire cysteine synthesis pathway starting from homoserine (Figure 5).

In addition to this pathway, a cysteine synthase gene (AO090102000276) was found to be upregulated during growth on PAC (log_2_FC = 1.45). This enzyme is part of a second cysteine synthesis route that originates from serine. In the reaction step catalyzed by this enzyme, O-acetyl-L-serine reacts with sulfide under the simultaneous cleavage of acetate to form cysteine. However, since we used sulfate as the sole sulfur source in our media, a multi-step reduction to sulfide had to occur prior to cysteine synthesis [80]. For this sulfate reduction, we were also able to identify some differentially expressed genes in our data sets (Figure 5). Compared with glucose, more genes were upregulated on pure acetate than on PAC, since the latter did not show an altered expression of the sulfate adenylyltransferase gene, catalyzing the reaction of sulfate to adenosine 5′-phosphosulfate (APS). A possible explanation for this observation could be that the PAC component furfural inhibits sulfur metabolism prior to its assimilation into cysteines, as reported for *E. coli*. Moreover, it was described in this study that the sulfur limitation caused by furfural led to the increased expression of genes involved in methionine and cysteine biosynthesis [81], which is consistent with the slightly higher fold changes in our PAC cultures.

Both cysteine and methionine are considered targets for oxidation by reactive oxygen species (ROS) in proteins [82]. Furthermore, although most ROS are generated as an inevitable consequence of cellular metabolism during aerobic growth, additional oxidative stress can also be caused by external environmental factors. For example, it was shown for *S. cerevisiae* that the addition of acetate to the growth medium caused a significant increase in superoxide dismutase and catalase activity, two enzymes that are responsible for the direct elimination of ROS [37]. In our data, we also observed the significant upregulation of genes encoding superoxide dismutase and also cytochrome c peroxidases, for both PAC and acetate (Table 4). In contrast, the transcriptional regulation of catalases was not that clear, as one gene (AO090701000158) was also found to be upregulated on glucose. However, this gene encodes for catalase A, whose expression in *A. oryzae* has been shown not to be altered by exposure to hydrogen peroxide (H_2_O_2_) [83].

Besides these enzymes of the primary antioxidant defense, there is also a non-enzymatic mechanism for the elimination of oxidative stress involving glutathione [84]. The addition of this compound to *C. glutamicum* shake flask cultures containing PAC from the bioliq^®^ plant at KIT has already been reported to counteract the growth-inhibiting effect of the condensate [85]. Glutathione is a tripeptide composed of glutamate, glycine and cysteine. Thus, the higher expression of genes needed for the synthesis of the latter amino acid could also be due to an increased demand for glutathione during growth in the acetate-containing media. For example, it has been shown for *Candida utilis* that not only ROS but also osmotic stress caused by high NaCl concentrations leads to increased glutathione synthesis [86], and, for *S. cerevisiae,* it was reported that enhanced cysteine synthesis results in higher tolerance to osmotic stress [87]. Consistent with the results of these studies, we found the upregulation of several genes related to the glutathione system in our cultures with PAC and pure acetate (Table 4).

In addition to glutathione synthase, these genes encode for enzymes such as glutathione S-transferases (GST) but also gluta- and thioredoxins. While GSTs are involved in the detoxification of xenobiotics via the conjugation of the foreign compounds with the sulfhydryl group of glutathione [88], the latter are mainly responsible for the repair of ROS-induced damage to cysteine residues in proteins [89,90]. However, some (monothiol) glutaredoxins also appear to be involved in the maintenance of iron homeostasis, as well as the synthesis of iron/sulfur clusters [91,92,93]. Since the respiratory chain includes numerous proteins containing such clusters and since oxidative phosphorylation was upregulated in the PAC cultures, the increased expression of glutaredoxin 3, as well as monothiol glutaredoxin, during fungal growth on the pyrolytic condensate seems reasonable. We also identified a few differentially expressed thioredoxin genes during growth on the different C-sources. One of these genes appeared to be strongly downregulated exclusively between pure acetate and PAC (AO090026000065), whereas another (AO090026000708) additionally showed decreased expression when comparing glucose and the pyrolytic condensate. However, one gene was also found to be upregulated in glucose, suggesting that oxidative stress may also occur under normal aerobic growth conditions.

The transcriptional regulation of GST genes also proved to be quite ambiguous under our experimental conditions (Table 4). It was expected that inhibitory PAC components might induce the increased expression of these enzymes, as this has recently been observed in *S. cerevisiae* in response to the addition of furfural and HMF [94]. Indeed, we were able to identify two GST genes (AO090003000631 and AO090103000485) that were upregulated on PAC compared to glucose and pure acetate. However, there were also some genes that showed higher expression levels during growth on glucose. Of particular note is the gene AO090103000149, for which a 5-fold and almost 10-fold increased expression was observed when glucose was compared with PAC and acetate, respectively. This observation may possibly be explained by the fact that GSTs have been ascribed several additional functions in the metabolism of endogenous compounds [88]. For example, the GST that was strongly upregulated in the glucose cultures had 47% similarity to URE2 in *S. cerevisiae*, a protein that was reported to function as a regulator in nitrogen metabolism [95]. Moreover, the possible involvement of GST in aflatoxin production of filamentous fungi has been suggested for *A. flavus* [96]. It would be conceivable that GSTs are also involved in the formation of other mycotoxins and that the upregulation of some GST genes during growth on glucose may therefore be associated with the increased expression of mycotoxin clusters in these cultures (Supplementary Table S2).

Despite the rather unclear transcriptional regulation of GST, our results generally indicate that cultivation of the fungus in both acetate-containing media caused osmotic and oxidative stress, which led to the upregulation of genes involved in their defense. Since the molecules involved in these defense mechanisms often rely on cysteines, this was probably the main reason for the increased expression of the cysteine-synthesis pathway, as well as the closely related sulfur metabolism.

##### Tryptophan Metabolism

**Figure 6:**
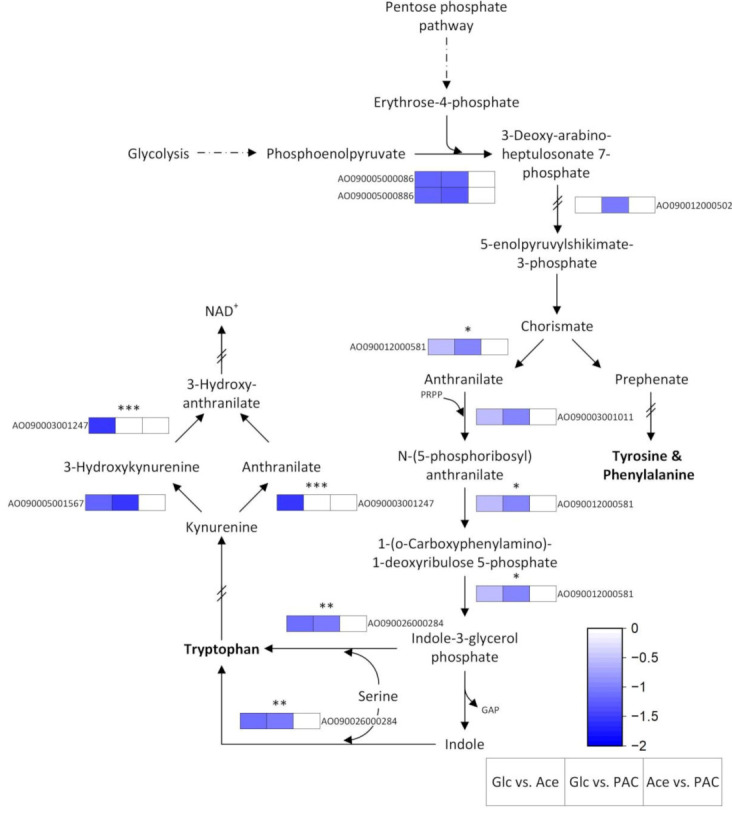
Differentially expressed genes of tryptophan metabolism during the cultivation of *A. oryzae* on glucose (Glc), acetate (Ace) and the pyrolytic aqueous condensate (PAC). Genes marked with asterisks (*, **, ***) code for enzymes involved in multiple reactions.

In addition to the significant upregulation of the cysteine and methionine metabolism, we also found an increased expression of numerous genes of tryptophan metabolism in our acetate-containing cultures (Table 2). This observation is consistent with previous reports in which *S. cerevisiae* showed an upregulation of tryptophan metabolism after the addition of acetate [97]. However, in our data, differential expression already started several reaction steps earlier, since genes of the shikimate pathway were found to be overexpressed in the acetate-containing cultures (Figure 6).

This pathway represents the first common part in the synthesis of all three aromatic amino acids and starts with the reaction of phosphoenolpyruvate and erythrose-4-phosphate to form 3-deoxy-arabino-heptulosonic acid 7-phosphate (DAHP). This first step is catalyzed by DAHP synthase, which is encoded by several genes in *A. oryzae*. Two of these genes (AO090005000086, AO090005000886) were found to be upregulated in both acetate and PAC when compared to growth on glucose. In the fungus, the majority of the subsequent reaction steps involved in the shikimate pathway require the participation of a common enzyme complex termed pentafunctional AROM polypeptide, whose gene (AO090012000502) was exclusively upregulated in PAC relative to glucose (log_2_FC = 1.08). After the final reaction step catalyzed by this complex, 5-enolpyruvylshikimate-3-phosphate is formed, which is then further converted to chorismate by chorismate synthase. Although the latter enzyme was not differentially expressed under any of the conditions tested, an increased requirement for chorismate, especially in the PAC-containing cultures, seems quite plausible, since the compound serves as a precursor for the synthesis of ubiquinone. This molecule, in turn, represents a key electron carrier in oxidative phosphorylation, a pathway that was found to be upregulated during fungal growth on the pyrolysis condensate (Table 2). In addition to the route of chorismate formation, we were also able to identify several differentially expressed genes involved in the further reaction steps of ubiquinone synthesis (Supplementary Table S2), reinforcing the hypothesis of the enhanced requirement for the electron carrier.

After the formation of chorismate, the synthesis pathway of the aromatic amino acids divides into prephenate, which is a precursor of phenylalanine and tyrosine, and anthranilate, from which tryptophan is formed in several further reaction steps. Three genes associated with tryptophan synthesis showed significant overrepresentation (FDR < 0.05), with log_2_FC values ranging between 1.16 and 0.54, in the cultures grown on acetate and PAC compared to the glucose control (Supplementary Table S2). Of these, the gene encoding tryptophan synthase, which catalyzes the final reaction step of indole-3-glycerol phosphate and serine to tryptophan, showed the highest increase (log_2_FC = 1.16). Thus, this observation may provide a further explanation for the abovementioned enhanced expression of genes involved in serine metabolism.

Although the underlying mechanism is still poorly understood, there are a number of studies showing that tryptophan plays a role in mitigating the effects of various types of stress, such as DNA damage [98] and, in particular, cell membrane stress. For example, the presence of the wild-type phosphoribosylanthranilate isomerase gene TRP1 in *S. cerevisiae* strains counteracted growth defects caused by the cell membrane disrupting detergent SDS [99]. Ethanol is also known for its negative effects on the cell membrane of microorganisms, as it causes disorganization in membrane structures and increases its permeability [100]. Accordingly, in a phenotypic analysis of *S. cerevisiae* single-gene-deletion strains, especially those strains with knock-outs in tryptophan metabolism, revealed increased ethanol sensitivity [101]. Interestingly, Yoshikawa et al. showed the occurrence of distinct phenotypes that were alcohol-specific and deletions that also resulted in growth deficits at high NaCl concentrations. It was revealed that knock-outs of genes involved in the synthesis of the precursor chorismate led to increased sensitivity to both stressors, whereas the majority of tryptophan-specific genes only caused a reduced tolerance to ethanol stress. This is contrary to our observation that the increased expression of the tryptophan synthase gene also occurred in the pure acetate cultures. 

Our findings are further supported by the study of Bauer et al. in which tryptophan was shown to play a role in the response to stress caused by weak acids such as acetate. In this work, tryptophan auxotrophic *S. cerevisiae* strains exhibited hypersensitivity to the acid, which could be recovered by supplementation with external tryptophan [46]. Moreover, it was stated in this study that weak acid stress inhibits the uptake of aromatic amino acids from the medium. This appears to be consistent with the results of our functional enrichment analysis, wherein we found that several amino acid transporters are downregulated in both acetate-containing cultures. Among them was also the homologue (AO090005000114) with the highest amino acid similarity (52% identity and 68% positives) to TAT2 in *S. cerevisiae*. TAT2 encodes for a tryptophan permease whose overexpression has been reported to reduce cellular sensitivity towards several types of stresses [99,102,103]. We found that the putative TAT2 gene was upregulated on glucose compared to pure acetate (log_2_FC = 2.71) and PAC (log_2_FC = 5.38). Moreover, the gene was also overexpressed in the comparison of acetate and PAC (log_2_FC = 2.67) and might therefore be a potential candidate for the genetic modification of *A. oryzae* to further increase its PAC tolerance.

However, in addition to genes related to tryptophan synthesis and transport, we also found some genes of the so-called kynurenine pathway to be differentially expressed (Figure 6). This pathway represents the central metabolic route of tryptophan degradation and is required for the *de novo* synthesis of NAD. The upregulation of such genes by acetate has been reported previously for *S. cerevisiae* [97] and may indicate a higher requirement for reduction equivalents in the acetate-containing cultures.

#### 3.4.3. Pyruvate Metabolism as a Key Pathway in the Comparison of Pure Acetate and PAC

**Figure 7:**
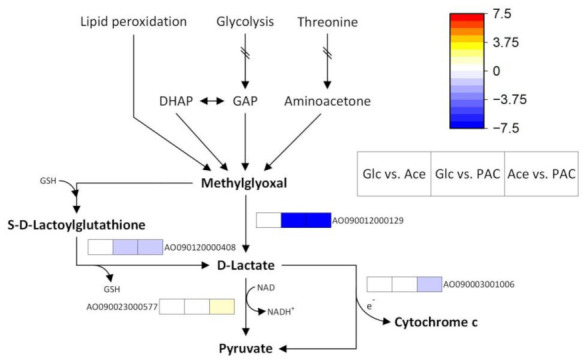
Differentially expressed genes of pyruvate metabolism during the cultivation of *A. oryzae* on glucose (Glc), acetate (Ace) and the pyrolytic aqueous condensate (PAC). DHAP = dihydroxyacetone phosphate; GAP = glyceraldehyde-3-phosphate.

Pathway analysis for the comparison of acetate and the pyrolysis condensate revealed that pyruvate metabolism was the only pathway that showed significant differences in transcriptional regulation. The differentially expressed genes associated with this pathway mainly included those for the degradation of methylglyoxal (MG) and several alcohol dehydrogenases (Figure 7). The former are of particular interest as MG is a reactive electrophile that is capable of causing damage to proteins and nucleotides [104].

In the cell, the oxoaldehyde is mainly formed from triose phosphate intermediates of glycolysis either by a non-enzymatic mechanism [105] or with the participation of the enzyme methylglyoxal synthase [106]. However, to the best of the authors’ knowledge, the presence of this enzyme has not yet been described for *A. oryzae*. In fact, the only glycolysis gene that we found to be upregulated in the PAC cultures encoded for fructose-bisphosphate aldolase (AO090009000324, log_2_FC = 1.57), an enzyme that catalyzes the synthesis of the triosephosphate DHAP. Thus, its increased expression might indicate enhanced MG formation from glycolysis intermediates in response to the presence of the pyrolysis condensate. However, lipid peroxidation [107] and threonine degradation [108] have also been reported as possible pathways for the formation of the oxoaldehyde. Moreover, it is also conceivable that the MG in the PAC cultures was derived from acetol, since this ketone is the major PAC component (Table A1) and the potential of *A. oryzae* to metabolize acetol has recently been reported [4]. However, the underlying pathway is still unclear, as the literature focused on the reverse reaction of the MG degradation to acetol or lactaldehyde. There are various enzymes capable of performing this conversion using NADH or NADPH as cofactors [109], but to date there are no studies reporting their reversibility.

Due to the high cytotoxicity of MG, there are further degradation mechanisms to prevent its accumulation and the resulting cellular damage. Probably the most well-known is the glyoxalase system, which has also been partly described in *A. niger* [110]. This system consists of two enzymatic steps, with glyoxalase I first catalyzing the condensation reaction of MG and glutathione. Subsequently, the resulting S-D-lactoylglutathione is hydrolyzed to D-lactic acid by glyoxalase II, recovering the glutathione (Figure 7). Accordingly, this mechanism represents a link between MG degradation and oxidative stress defense, and its increased expression may provide an additional explanation for the upregulation of genes related to the glutathione system and cysteine synthesis.

The comparison of pure acetate and PAC revealed the upregulation of glyoxalase II (log_2_FC = 1.05) in the cultures containing the pyrolysis condensate, whereas no significant difference was detected for glyoxalase I. The latter observation could possibly be explained by the fact that glyoxalase I was reported to be induced by osmotic stress [111], a condition that was present in both cultures. By contrast, the upregulation of glyoxalase II during growth on PAC may be related to the increased expression of oxidative phosphorylation genes in these cultures (Table 2). D-lactate formed by glyoxalase II can be converted to pyruvate by a mitochondrial D-lactate dehydrogenase (D-LDH). During this reaction, electrons are transferred to cytochrome c, thus coupling MG degradation and cellular respiration. Accordingly, the oxidation of D-lactate under oxidative stress conditions can drive the respiratory chain without involving complex I, as this is considered one of the major sites of mitochondrial ROS production [112]. In our PAC cultures, we found a gene encoding cytochrome c-linked D-LDH to be upregulated compared to pure acetate (AO090003001006; log_2_FC = 1.75), whereas a NADH-dependent D-LDH gene showed decreased expression (AO090023000577; log_2_FC = −1.11) (Supplementary Table S2).

However, an even more significant differential expression was observed for another MG degradation pathway that also leads to the formation of D-lactate. This pathway has already been described for yeasts [113,114] and involves only a single glutathione-independent reaction catalyzed by another glyoxalase (Figure 7). For the gene encoding this enzyme (AO090012000129), our data showed strong upregulation in the PAC-containing cultures compared with both pure acetate (log_2_FC = 7.49) and glucose (log_2_FC = 6.63), indicating that the glutathione-independent reaction appears to be the main MG-degradation pathway during growth on PAC. This raised the question of whether cellular glutathione levels were insufficient to compensate for the amount of MG produced and whether a deficiency of this thiol compound may have occurred. In addition to the aforementioned upregulation of the glutathione synthase gene, this assumption might also be supported by the differential expression of alcohol dehydrogenases in our cultures (Table 5).

For example, it has been observed that, in glutathione-depleted *Candida albicans* strains, ADH1 functions as methylglyoxal reductase and catalyzes the oxidation of MG to pyruvate [115]. Moreover, the reduction of the oxoaldehyde to acetol can also be performed by this enzyme [115,116]. However, the ADHs involved in these reactions were NADH-dependent, whereas in our PAC-containing cultures we rather found an increased expression of ADHs that require NAPDH as a cofactor (Table 5). Among these, the genes AO090010000668 and AO090023000460 might be of particular interest. While the former showed remarkably high expression compared to both glucose (log_2_FC = 6.14) and acetate (log_2_FC = 8.02), the latter was differentially expressed exclusively between acetate and PAC, and the altered gene expression can therefore probably be attributed to additional PAC components.

Furan compounds like 5-hydroxymethylfurfural (HMF) and furfural might represent such components, since their microbial conversion into less-harmful alcohols has been reported to involve the action of ADHs in *S. cerevisiae* [117,118]. In particular, the NADPH-preferring ADH6 was characterized by its high in vitro activity towards the two furans, but, in addition, the overexpression of the corresponding gene led to an increase in growth and the HMF-uptake rate in anaerobic batch cultivation [118]. Conversely, an inhibitory effect of furfural towards NADH-dependent ADH (EC 1.1.1.1) has been described by Modig et al. [119], which could partly explain the observed downregulation of these genes in the PAC-containing cultures. In addition, there is a study on *S. cerevisiae* showing that carbon catabolite repression in microorganisms is not limited to glucose but also that growth on acetate can lead to a decreased expression of alcohol dehydrogenases like ADH2 [120].

Besides the NAPDH-dependent ADHs, a gene encoding for a so-called “old yellow enzyme” (OYE) also showed strongly increased expression on PAC in comparison to acetate and glucose (AO090005001535; log_2_FC_Acetate_ = 7.81, log_2_FC_Glucose_ = 4.81). These oxidoreductases may be of particular interest for further improving fungal PAC tolerance, as they catalyze the NADPH-dependent reduction of α, β-carbon double bonds in a variety of substrates [121]. Since these potential substrates also include cyclic components, it is quite conceivable that the enzyme is capable of converting 2-cyclopenten-1-one, a PAC component that is highly toxic to *A. oryzae* [10], into the less harmful alkane. However, the other genes encoding enzymes of MG degradation and NAPDH-dependent ADHs described in this section may also be potential targets for a strain engineering aimed at increasing fungal PAC tolerance.

## 4. Conclusions

In summary, this study allowed us to gain comprehensive insights into the transcriptional response of *A. oryzae* to growth on detoxified pyrolytic aqueous condensate (PAC) and compare it to acetate and glucose. High similarity in the transcriptome profiles of PAC and pure acetate occurred, likely, due to the high acetate content in the former. For example, functional enrichment analysis revealed the upregulation of GO terms associated with ribosomes and non-coding RNA processing in both cultures, whereas transmembrane transport was downregulated. Among the identified transporter genes were mainly those of the major facilitator superfamily, including sugar transporters and several transporters involved in the secretion of secondary metabolites (SM). Closer examination of the associated SM gene clusters revealed a decreased expression of the genes encoding kojic acid, heptelidic acid and cyclopiazonic acid, especially in PAC cultures. Accordingly, PAC components appear to influence the biosynthesis of some SM, a finding that may be of interest when studying targeted natural product synthesis.

Our analysis also revealed significant variation in the expression of carbon metabolism and the biosynthesis of amino acids among the compared C-sources. While the central carbon metabolism during growth on the monosaccharide mainly involved glycolysis/gluconeogenesis, as well as starch and sucrose metabolism, 2-oxocarboxylic acid metabolism was the most abundant pathway in the acetate-containing cultures. Moreover, the expression of genes associated with the glyoxylate pathway was significantly increased in *A. oryzae* grown on pure acetate, whereas cultivation in a PAC-containing medium additionally resulted in the enhanced transcription of genes involved in the TCA cycle and the closely related oxidative phosphorylation. We further observed the preponderance of cysteine and methionine metabolism, as well as tryptophan metabolism, among the upregulated amino acid biosynthesis pathways in both acetate-containing cultures. In addition, the increased synthesis of serine- and arginine-associated genes suggest that these amino acids also play a role in the transcriptional response to growth on these substrates. While arginine is mainly known for its protective effect against osmotic stressors, enhanced cysteine expression is primarily associated with oxidative stress. The presence of oxidative stress was also confirmed by the upregulation of several genes related to the glutathione system, suggesting that growth on acetate-containing media evokes an extensive stress response.

Despite many similarities in the transcriptome of the fungus during growth on acetate and PAC, a direct comparison of the two substrates showed that the expression of pyruvate metabolism differed significantly. The pathway was overexpressed in the PAC cultures and mainly included genes associated with the degradation of methylglyoxal and numerous alcohol dehydrogenases. In addition, a gene encoding an “old yellow enzyme” was among the most expressed genes in the PAC cultures. This gene is of particular interest because it may be able to degrade 2-cyclopenten-1-one, one of the most-toxic PAC components, to *A. oryzae*. However, several other differentially expressed genes implicated in amino acid synthesis and stress response are also highly relevant as potential targets for increasing fungal PAC tolerance. Future studies are now required to examine whether the observed variations in gene expression result in actual changes in the fungal metabolome and if targeted overexpression of these genes will generate moreresistant *A. oryzae* strains.

## Figures and Tables

**Table 1:** Quality characteristics of the RNA-seq data obtained for *A. oryzae* cultivated on glucose, acetate and 20% detoxified PAC.

Sample	Read Count		Alignment Ratio [%]	
	Raw	Clean	Uniquely Mapped	Unaligned
Glc1	22,221,898	17,902,548	97	3
Glc2	20,409,071	16,336,686	96	3
Glc3	21,015,522	16,346,846	93	6
Ace1	19,792,462	15,781,442	95	4
Ace2	18,718,483	14,332,714	93	7
Ace3	20,242,931	15,735,543	93	7
PAC1	22,870,039	17,628,982	94	5
PAC2	19,884,644	15,059,444	96	4
PAC3	19,141,036	14,198,088	86	14

**Table 2:** Differentially expressed metabolic pathways for the comparisons of glucose with pure acetate and PAC.

Condition	DGE	KEGG Pathway ID	KEGG Pathway Name	Set Size	Adjusted *p*-Value
Glc vs. Ace	up	aor00500	Starch and sucrose metabolism	18	4.12 × 10^−4^
		aor00010	Glycolysis/Gluconeogenesis	14	2.61 × 10^−2^
	down	aor03008	Ribosome biogenesis in eukaryotes	27	7.72 × 10^−14^
		aor01230	Biosynthesis of amino acids	39	4.10 × 10^−8^
		aor01210	2-oxocarboxylic acid metabolism	12	4.12 × 10^−4^
		aor01200	Carbon metabolism	32	4.12 × 10^−4^
		aor01110	Biosynthesis of secondary metabolites	126	4.76 × 10^−4^
		aor00270	Cysteine and methionine metabolism	11	5.20 × 10^−4^
		aor01100	Metabolic pathways	253	1.23 × 10^−3^
		aor00380	Tryptophan metabolism	16	4.14 × 10^−3^
		aor00230	Purine metabolism	16	9.92 × 10^−3^
		aor00630	Glyoxylate and dicarboxylate metabolism	12	0.01
		aor00360	Phenylalanine metabolism	11	0.02
Glc vs. PAC	up	aor00500	Starch and sucrose metabolism	25	1.50 × 10^−4^
		aor00520	Amino sugar and nucleotide sugar metabolism	19	4.40 × 10^−3^
		aor00100	Steroid biosynthesis	13	0.01
	down	aor03008	Ribosome biogenesis in eukaryotes	40	1.46 × 10^−15^
		aor03010	Ribosome	39	8.16 × 10^−14^
		aor01230	Biosynthesis of amino acids	61	1.03 × 10^−11^
		aor01210	2-oxocarboxylic acid metabolism	25	5.34 × 10^−9^
		aor03013	RNA transport	25	6.75 × 10^−9^
		aor01200	Carbon metabolism	51	1.16 × 10^−7^
		aor00270	Cysteine and methionine metabolism	19	1.50 × 10^−6^
		aor00970	Aminoacyl-tRNA biosynthesis	19	2.78 × 10^−6^
		aor03040	Spliceosome	16	3.68 × 10^−6^
		aor01110	Biosynthesis of secondary metabolites	182	1.22 × 10^−5^
		aor03020	RNA polymerase	12	1.31 × 10^−5^
		aor00190	Oxidative phosphorylation	16	2.23 × 10^−5^
		aor01100	Metabolic pathways	365	2.98 × 10^−5^
		aor03018	RNA degradation	18	5.57 × 10^−5^
		aor00020	Citrate cycle (TCA cycle)	16	8.90 × 10^−5^
		aor00290	Valine, leucin and isoleucine biosynthesis	11	1.22 × 10^−3^
		aor00630	Glyoxylate and dicarboxylate metabolism	20	1.27 × 10^−3^
		aor00680	Methane metabolism	11	2.18 × 10^−3^
		aor00250	Alanine, aspartate and glutamate metabolism	15	8.81 × 10^−3^
		aor00380	Tryptophan metabolism	20	9.44 × 10^−3^
		aor00620	Pyruvate metabolism	26	0.01
		aor00770	Pantothenate and CoA biosynthesis	15	0.02
		aor01212	Fatty acid metabolism	14	0.03
		aor00230	Purine metabolism	20	0.03

**Table 3:** Differentially expressed genes involved in the “starch and sucrose metabolism”.

Enzyme	E.C.	Regulation	Gene ID	Fold Change	
				Glc vs. Ace	Glc vs. PAC
β-fructofuranosidase	3.2.1.26	up	AO090020000640	1.71	2.71
			AO090701000038	1.51	1.50
α-glucosidase	3.2.1.20		AO090003001209	4.18	3.93
trehalose 6-phosphate (T6P) synthase	2.4.1.15 2.4.1.347		AO090102000159	3.83	3.39
T6P synthase/phosphatase subunit	2.4.1.15 3.1.3.12		AO090005001531	1.40	1.46
glucan endo-1,3-β-D-glucosidase	3.2.1.39		AO090009000117	1.24	1.13
glucan 1,3-β-glucosidase	3.2.1.58		AO090011000362	1.39	-
			AO090001000604	-	1.53
			AO090003000990	-	3.11
β-glucosidase	3.2.1.21		AO090001000544	1.42	1.80
			AO090038000425	2.37	-
			AO090003000497	-	1.97
			AO090001000266	-	3.66
			AO090701000274	-	1.12
endoglucanase	3.2.1.4		AO090011000715	2.34	2.54
			AO090003001342	-	3.70
arabinogalactan endo-1,4-β-galactosidase	3.2.1.89		AO090001000492	2.04	-
cellulose 1,4-β-cellobiosidase	3.2.1.91		AO090001000348	2.16	3.95
glycogen debranching enzyme	2.4.1.25 3.2.1.33		AO090005000884	1.43	1.79
α-amylase	3.2.1.1		AO090003001497	4.19	2.50
			AO090003001498	2.24	1.20
			AO090023000944	6.33	7.45
			AO090120000196	6.40	7.07
glucoamylase	3.2.1.3		AO090010000746	6.63	9.13
1,4-α-glucan branching enzyme	2.4.1.18		AO090010000483	1.02	1.66
1,3-β-glucan synthase	2.4.1.34		AO090009000174	-	1.11
glucan 1,3-β-glucosidase	3.2.1.58	down	AO090038000279	-	−1.04
β-glucosidase	3.2.1.21		AO090701000841	-	−3.24

**Table 4:** Transcriptional regulation of genes involved in the oxidative-stress defense.

Enzyme	EC	Gene ID	Fold Change		
			Glc vs. Ace	Glc vs. PAC	Ace vs. PAC
catalase	1.11.1.6	AO090701000158	2.38	2.76	-
		AO090120000068	−1.15	-	1.07
		AO090020000389	-	−6.43	−4.00
cytochrome c peroxidase	1.11.1.5	AO090023000654	−1.26	−1.48	-
		AO090103000329	−2.95	−2.67	-
superoxide dismutase	1.15.1.1	AO090005001580	−1.01	−1.60	-
		AO090020000521	−1.20	−1.30	
glutathione synthase	6.3.2.3	AO090701000193	-	−1.07	−0.96
glutaredoxin 3	-	AO090009000473	-	−1.17	−0.95
monothiol glutaredoxin	-	AO090023001002	-	−1.47	-
thioredoxin		AO090026000065	-	-	−5.80
		AO090026000708	-	−1.28	−1.40
		AO090020000504	2.05	1.78	-
glutathione S-transferase	2.5.1.18	AO090005000973	−1.36	−1.16	-
		AO090003000631	-	−2.87	−3.31
		AO090103000485	-	−2.73	−2.37
		AO090012000378	2.34	-	−2.51
		AO090010000447	-	1.18	-
		AO090103000134	1.49	1.79	-
		AO090103000149	9.85	5.37	−4.50

**Table 5:** Differentially expressed NADH- (EC 1.1.1.1) and NADPH-dependent (EC 1.1.1.2) alcohol dehydrogenases.

Condition	EC	Gene ID	Fold Change
Glc vs. PAC	1.1.1.1	AO090005000125	1.68
		AO090038000108	2.85
		AO090026000555	3.43
	1.1.1.2	AO090005001358	−3.78
		AO090010000668	−6.14
Glc vs. Ace	1.1.1.1	AO090009000634	1.47
		AO090038000108	4.19
	1.1.1.2	AO090038000575	−1.62
Ace vs. PAC	1.1.1.1	AO090026000555	2.28
	1.1.1.2	AO090005001358	−3.63
		AO090023000460	−3.39
		AO090010000668	−8.02

## Data Availability

The RNA-seq data underlying this study were uploaded to the NCBI Sequence Read Archive (SRA) database under the following BioProject ID: PRJNA845824.

## Supplementary Materials

The following supporting information can be downloaded at: https://www.mdpi.com/article/10.3390/jof8080765/s1, Table S1: Differentially expressed genes assigned to “transmembrane transport” (GO:0055085); Table S2: Summary of differentially expressed genes mentioned in this article; Supplementary Figure S1: Temporal change of pH value during preliminary *A. oryzae* shake-flask cultivation. The data are means of three biological replicates, and the error bars represent the standard deviation.

## Appendix A

**Table A1:** GC/MS analysis of the PAC composition before and after detoxification.

Component	Concentration [g/L]	
	Before Detoxification	After Detoxification
acetic acid	35.40	40.05 ^1^
propionic acid	10.42	0
possibly propanoic acid, ethenyl ester	0.18	0
ethylene glycol	3.27	7.63
hydroxy-acetaldehyde	1.90	0
3-hydroxy propionaldehyde	0.28	0
2-butenal	0.58	0
butandial (or propanal)	0.34	0
acetol (hydroxypropanone)	51.46	38.42
2-butanone	1.98	0.38
1-hydroxy-2-butanone	6.71	3.86
2,3- butandione (diacetyl)	2.40	0.37
3-hydroxy-2-butanone(acetoin)	1.42	1.54
1-acetyloxy-propan-2-one	1.07	0
cyclopentanone	1.12	0
2-cyclopenten-1-one	3.35	0.33
2,3-dimethyl-2-cyclopenten-1-one	0.22	0
2-methyl-2-cyclopenten-1-one	1.35	0
3-methyl-2-cyclopenten-1-one	0.68	0
2-cyclohexen-1-one	0.11	0
possibly 3-methyl-2-butanone	0.09	0
possibly 3-methyl-3-buten-2-one	0.16	0
2-pentanone	0.51	0
2,3-pentanedione	0.63	0.17
3-penten-2-one	0.44	0
isomere of 3-methyl-2-Cyclopenten-1-one	0.28	0
possibly 2-ethyl-butanal	0.31	0
possibly 3,4-dimethyl-2-cyclopenten-1-one	0.27	0
isomere of 2,3-dimethyl-2-cyclopenten-1-one	0.42	0
3-methyl-1,2-cyclopentanedione	1.50	0
2,3,4-trimethyl-2-cyclopenten-1-one	0.17	0
2(5H)-furanone	0.64	0
2-furaldehyde	2.84	0.16
5-methyl-2-furaldehyde	0.13	0
1-(2-furanyl)-ethanone	0.30	0
3-methyl-, (5H)-furan-2-one	0.04	0
γ-butyrolactone	0.71	0.71
1-methoxy-4-methyl-benzene	0.06	0
indene	0.08	0
phenol	0.63	0
o-cresol	0.46	0
p-cresol	0.17	0
m-cresol	0.21	0
2,5-dimethyl-phenol	0.19	0
2,4-dimethyl-phenol	0.08	0
2,6-dimethyl-phenol	0.06	0
4-ethyl-phenol	0.14	0
guaiacol	1.80	0
3-methyl-guaiacol	0.16	0
4-methyl-guaiacol	0.36	0
4-ethyl-guaiacol	0.27	0
4-vinyl-guaiacol	0.24	0
syringol	0.17	0
D-limonene or isomere	0.14	0
2-acetyl-5-norbornene	0.06	0
2-methyl-1,3-dioxolane	0.12	0
possibly 2,3-dihydro-1,4-dioxin	0.09	0

^1^ as already mentioned in [11], the acetate content of the PAC increases during the overliming treatment.

**Table A2:** Differentially expressed metabolic pathways for the comparisons of glucose with pure acetate and PAC that could not be considered significant.

Condition	DGE	KEGG Pathway ID	KEGG Pathway Name	Set Size	Adjusted *p*-Value
Glc vs. Ace	down	aor00330	Arginine and proline metabolism	14	0.06
		aor01212	Fatty acid metabolism	13	0.10
		aor00071	Fatty acid degradation	12	0.17
		aor00250	Alanine, aspartate and glutamate metabolism	14	0.17
		aor00260	Glycine, serine and threonine metabolism	12	0.20
Glc vs. PAC	up	aor02010	ABC transporters	10	0.09
		aor00564	Glycerophospholipid metabolism	16	0.17
	down	aor00260	Glycine, serine and threonine metabolism	19	0.06
		aor04146	Peroxisome	20	0.11
		aor00330	Arginine and proline metabolism	16	0.11

## Appendix B

### Differential Expression of Selected Secondary Metabolite Clusters

Since functional enrichment analysis revealed that several transporters involved in SM secretion were upregulated in the glucose cultures, the associated gene clusters will be investigated more intensively in the following section.

Probably the best-known SM produced by *A. oryzae* is kojic acid, the synthesis gene cluster of which comprises only three genes: *kojA*, *kojR* and *kojT* [122]. Among these genes, *kojR* was previously shown to serve as a transcription factor exerting an activating effect on the gene of FAD-dependent oxidoreductase (*kojA*), which is likely responsible for the actual kojic acid biosynthesis, and the MFS transporter (*kojT*) [44,123]. As indicated in Table A3, all three genes were upregulated in the glucose cultures, although for *kojT,* the increased expression was only observed in comparison with PAC. The upregulation of the kojic acid cluster during growth on glucose seems plausible, since it has been suggested that, unlike other SM, this compound can be synthesized directly from glucose [122].

Heptelidic acid (HA) is another secondary metabolite produced by *A. oryzae*, an antibiotic reported to inhibit halotolerant lactic acid bacteria during the second stage of soy sauce fermentation [124]. HA acts by irreversibly inactivating the gpd enzyme of bacteria via the covalent modification of a cysteine thiol in the active center of the protein [125,126]. It was therefore suggested that the *gpdB* (or *hepG*) gene contained in the HA cluster is involved in fungal self-resistance to this SM [125]. In addition to *hepG* and the MFS transporter gene *hepF*, the HA gene cluster includes a sesquiterpene synthase (*hepA*), four cytochrome P450 genes (*hepC*, *hepD*, *hepE* and *hepH*), a putative antibiotic biosynthesis monooxygenase (*hepB*) and two transcription factors (*hepR* and *hepS*) [43,125]. Table A3 shows that differential expression was found for all these genes except *hepB*, which is not predicted in the *A. oryzae* RIB40 genome. As with kojic acid, the two transcriptional regulator genes were upregulated only in glucose compared to PAC cultures, whereas the remaining genes also showed increased expression when the monosaccharide and acetate were compared. The fold changes were always lower for the latter comparison, indicating once again that the PAC components may cause additional inhibition. However, as far as the authors are aware, there are currently no studies addressing the effects of lignocellulosic inhibitors on fungal SM production.

**Table A3:** Selection of differentially expressed secondary metabolite clusters.

Secondary Metabolite	Gene Name	Gene ID	Fold Change		
			Glc vs. Ace	Glc vs. PAC	Ace vs. PAC
heptelidic acid	*hepA*	AO090011000408	4.82	8.09	3.28
	*hepB* ^1^	-	-	-	-
	*hepC*	AO090011000410	6.05	8.52	-
	*hepD*	AO090011000411	4.85	7.03	-
	*hepE*	AO090011000412	5.51	7.27	-
	*hepF*	AO090011000413	2.61	5.50	2.89
	*hepG (gpdB)*	AO090011000414	2.03	3.46	1.43
	*hepH*	AO090011000415	3.99	7.46	3.47
	*hepR*	AO090011000416	-	1.29	-
	*hepS*	AO090011000417	-	1.32	-
kojic acid	*kojA*	AO090113000136	1.67	2.11	-
	*kojR*	AO090113000137	2.13	2.95	-
	*kojT*	AO090113000138	-	2.05	-
cyclopiazonic acid	*cpaR* ^1^	-	-	-	-
	*cpaA*	AO090026000001	-	5.67	5.42
	*cpaD*	AO090026000002	-	6.88	7.56
	*cpaO*	AO090026000003	-	7.37	7.83
	*cpaH*	AO090026000004	1.45	9.43	7.98
	*cpaM*^2^/*cpaT*	AO090026000005	-	6.00	5.34

^1^ gene is not found in the genome annotation of *A. oryzae* RIB40 ^2^ according to [127], the coding region of *cpaM* is partially contained in AO090026000005.

Since the second stage of soy sauce fermentation involves the addition of salt water and osmotolerant lactic acid bacteria and yeast to the koji (*Aspergillus*) culture [43], the authors would have expected the high salinity in the acetate and PAC cultures to induce the expression of the HA gene cluster. Instead, upregulation during growth on glucose was observed, suggesting that there may be other triggers of expression. For instance, it is conceivable that HA synthesis was induced by the low pH in the glucose cultures (Supplementary Figure S1), since the lactic acid fermentation occurring during the soy sauce production process also causes a pH drop [128]. Another explanation could be that the HA precursor farnesyl pyrophosphate is formed from acetyl-CoA via the mevalonate pathway, and since the overexpression of the TCA and glyoxylate cycle genes in the two acetate-containing cultures indicates an increased flux through these pathways, less acetyl-CoA may be available for HA synthesis. This assumption is supported by the downregulation of some mevalonate pathway genes especially in the PAC cultures. (Supplementary Table S2).

Another SM that also requires acetyl-CoA as a precursor and for whose upregulation in the glucose cultures the same possible explanation might therefore apply is the mycotoxin cyclopiazonic acid (CPA). The annotation of the corresponding synthesis cluster in *A. oryzae* RIB40 comprises only six genes since, according to Kato et al., the *cpaR* gene encoding a C6 zinc finger transcription factor is absent [127]. Moreover, the polyketide synthase gene *cpaA* is severely truncated in this strain, which is probably the main reason for its non-toxigenicity [127]. We found highly significant differential expression of *cpaA*, as well as of all other genes of the cluster, that were annotated in *A. oryzae* RIB40 (Table A3). However, due to the incompleteness of this cluster in our reference strain, it is difficult to assess whether the observed expression in our cultures actually resulted in the formation of a functional SM. In contrast to HA, the transcription of genes involved in CPA synthesis seemed to be inhibited mainly by PAC, as the comparison between glucose and pure acetate showed only one putative cytochrome P450 gene (*cpaH*) to be upregulated. Since CPA is a mycotoxin [129], it might be of interest to investigate which PAC components caused the inhibition in the gene expression and whether such inhibitors might make the handling of mycotoxin-producing strains easier and safer.
