# Supplementary material for: RNA-Seq Based Transcriptome Analysis of Aspergillus oryzae DSM 1863 Grown on Glucose, Acetate and an Aqueous Condensate from the Fast Pyrolysis of Wheat Straw
Source: J Fungi (Basel). 2022 Jul 23;8(8):765. doi: 10.3390/jof8080765 (PMC9394295; doi:10.3390/jof8080765)
Supplement: Supplementary file 1 [file jof-08-00765-s001.zip › Figure S1.pdf]

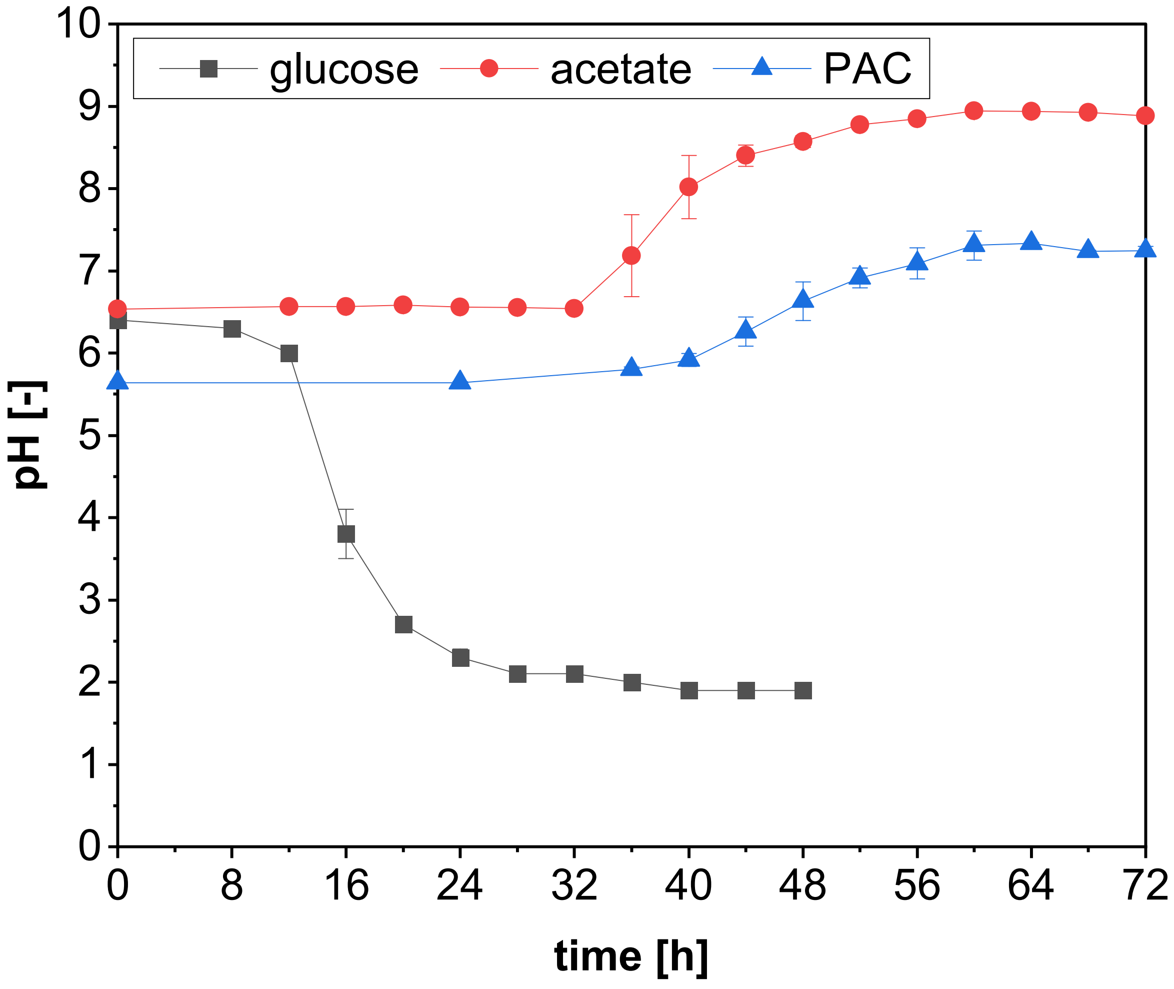

Figure S1: Temporal change of pH value during preliminary *A. oryzae* shake-flask cultivation. The data are means of three biological replicates, and the error bars represent the standard deviation.
