# Supplementary material for: RNA-Seq Based Transcriptome Analysis of Aspergillus oryzae DSM 1863 Grown on Glucose, Acetate and an Aqueous Condensate from the Fast Pyrolysis of Wheat Straw
Source: J Fungi (Basel). 2022 Jul 23;8(8):765. doi: 10.3390/jof8080765 (PMC9394295; doi:10.3390/jof8080765)
Supplement: Supplementary file 1 [file jof-08-00765-s001.zip › Table S1 Transporter genes.pdf]

Table S1: Differentially expressed genes assigned to “transmembrane transport” (GO:0055085)

Gene ID	Description	Fold change		
		Glc vs. Ace	Glc vs. PAC	Ace vs. PAC
AO090001000215	MFS allantoate transporter	2.28	2.61	-
AO090001000232	Belongs to the MFS. Proton-dependent oligopeptide transporter (POT/PTR) family (TC 2.A.17)	5.12	4.75	-
AO090001000305	Belongs to the MFS. Proton-dependent oligopeptide transporter (POT/PTR) family (TC 2.A.17)	3.90	3.29	-
AO090001000313	Multidrug resistance-associated protein	4.59	6.22	-
AO090001000360	Major Facilitator Superfamily	1.87	1.58	-
AO090001000404	MFS transporter	2.41	3.84	-
AO090001000692	Predicted transporter	1.23	1.49	-
AO090001000707	Ammonium transporter	1.65	2.15	-
AO090003000044	MFS transporter	1.68	2.46	-
AO090003000227	Fungal potassium channel	1.55	1.93	-
AO090003000268	Belongs to the purine-cytosine permease family (2.A.39)	3.84	3.27	-
AO090003000529	Vacuolar iron transporter Ccc1	2.06	-	-
AO090003000614	Calcium-transporting ATPase	1.85	-1.92	-3.77
AO090003000854	Urea transporter; belongs to the sodium solute symporter (SSF) family (TC 2.A.21)	2.71	5.25	-
AO090003000971	MFS transporter	1.21	1.78	-
AO090003001015	Belongs to the major facilitator superfamily. Sugar transporter family (TC 2.A.1.1)	2.35	3.23	-
AO090003001191	Mitochondrial calcium uniporter	2.34	1.72	-
AO090003001260	MFS transporter	4.26	6.13	-
AO090003001277	Belongs to the major facilitator superfamily. Sugar transporter family (TC 2.A.1.1)	1.37	-	-
AO090003001296	Belongs to the ZIP transporter family (TC 2.A.5)	3.27	8.48	5.22
AO090003001428	Efflux pump antibiotic resistance protein	4.75	7.98	3.23
AO090003001436	amino acid transporter	2.57	4.03	-
AO090003001482	Small oligopeptide transporter, OPT family	10.53	8.79	-
AO090003001549	Belongs to the MFS. Proton-dependent oligopeptide transporter (POT/PTR) family (TC 2.A.17)	3.76	6.23	2.47
AO090005000060	amino acid transporter	1.66	6.68	5.03
AO090005000114	Amino acid permease (Gap1)	2.71	5.38	2.67
AO090005000166	Putative siderochrome-iron transporter	1.77	1.90	-
AO090005000295	Major Facilitator Superfamily	1.02	2.18	1.16
AO090005000420	Sugar transporter family protein	4.41	5.06	-

Gene ID	Description	Fold change		
		Glc vs. Ace	Glc vs. PAC	Ace vs. PAC
AO090005000455	purine permease	1.72	2.22	-
AO090005000535	Major Facilitator Superfamily	9.01	7.68	-
AO090005000536	K+/H+-antiporter	1.33	1.21	-
AO090005000633	MFS transporter	1.24	1.01	-
AO090005000649	Amino acid permease	2.16	1.50	-
AO090005000863	Putative MFS transporter	1.19	2.15	-
AO090005001095	Auxin Efflux Carrier superfamily	1.20	1.27	-
AO090005001212	Predicted amino acid transporter	1.81	2.02	-
AO090005001328	Fungal trichothecene efflux pump (TRI12)	3.34	5.95	-
AO090005001343	Peptide transporter MTD1	1.96	4.50	2.54
AO090009000008	Sugar (and other) transporter	2.70	1.46	-
AO090009000479	Integral membrane protein	1.03	1.12	-
AO090009000565	Multidrug resistance-associated protein mitoxantrone resistance protein	3.76	-	-
AO090009000584	Major Facilitator Superfamily	2.47	2.50	-
AO090009000635	amino acid transporter	1.58	2.07	-
AO090009000651	multidrug/pheromone exporter, ABC superfamily	1.35	1.35	-
AO090010000024	Belongs to the MIP aquaporin family (TC 1.A.8)	3.05	2.10	-
AO090010000062	amino acid transporter	1.68	1.57	-
AO090010000119	AAT family amino acid transporter	2.17	3.36	-
AO090010000135	Iron-regulated transporter	6.76	4.89	-
AO090010000142	MFS transporter	2.18	-	-2.14
AO090010000195	Belongs to the MFS. Sugar transporter family (TC 2.A.1.1)	2.03	-	-
AO090010000219	ABC multidrug transporter atrA	1.40	2.20	-
AO090010000221	Bacteriorhodopsin-like protein	3.96	5.09	-
AO090010000222	Belongs to the MFS	2.47	3.49	-
AO090010000345	Permease for cytosine/purines, uracil, thiamine, allantoin	3.92	9.42	5.51
AO090010000639	Belongs to the MFS	4.66	-8.74	-13.40
AO090010000652	MFS transporter	1.79	2.68	-
AO090010000674	Permease of the MFS	1.47	2.40	-
AO090010000705	Belongs to the MIP aquaporin family (TC 1.A.8)	1.67	1.59	-
AO090010000723	Belongs to the MFS	2.31	6.99	4.68
AO090010000733	Sugar (and other) transporter	3.20	3.19	-
AO090011000151	Belongs to the MFS	5.14	-	-
AO090011000212	Low affinity iron transporter	7.08	6.13	-
AO090011000335	MFS drug transporter	5.83	5.25	-

Gene ID	Description	Fold change		
		Glc vs. Ace	Glc vs. PAC	Ace vs. PAC
AO090011000363	Cation diffusion facilitator 1	1.23	1.48	-
AO090011000378	ABC multidrug transporter atrG	1.59	1.07	-
AO090011000413	MFS-type transporter hepF	2.61	5.50	2.89
AO090011000587	MFS transporter	1.85	2.03	-
AO090011000649	Belongs to the purine-cytosine permease family (2.A.39)	2.53	3.10	-
AO090011000734	Belongs to the TrkH potassium transport family	2.29	-	-
AO090011000744	Carboxylic acid transport protein	3.37	-	-
AO090012000284	Belongs to the MFS. Sugar transporter family (TC 2.A.1.1)	2.13	1.13	-
AO090012000288	Belongs to the MFS	1.55	1.66	-
AO090012000289	Vacuole effluxer Atg22 like	1.06	-	-1.03
AO090012000315	Small oligopeptide transporter, OPT family	5.09	11.48	6.39
AO090012000494	Putative MFS transporter	1.15	1.08	-
AO090012000565	Uridine permease	3.29	5.56	-
AO090012000623	Nitrate transporter of the MFS	7.14	6.56	-
AO090012000710	Belongs to the MFS	3.99	3.63	-
AO090012000732	Belongs to the MFS. Sugar transporter family (TC 2.A.1.1)	1.51	2.08	-
AO090012000758	Belongs to the MFS	1.86	-	-
AO090020000209	Belongs to the MFS. Sugar transporter family (TC 2.A.1.1)	2.10	1.38	-
AO090020000236	Sugar (and other) transporter	5.69	6.07	-
AO090020000259	Belongs to the MFS. Sugar transporter family (TC 2.A.1.1)	1.53	2.05	-
AO090010000229	MFS peptide transporter Ptr2	-	1.05	1.12
AO090020000280	Predicted allantoate permease	1.26	1.43	-
AO090020000524	Belongs to the MFS. Proton-dependent oligopeptide transporter (POT/PTR) family (TC 2.A.17)	1.68	2.36	-
AO090020000533	Predicted protein	5.20	7.80	-
AO090020000639	Belongs to the MFS. Sugar transporter family (TC 2.A.1.1)	4.25	6.51	-
AO090020000668	Belongs to the MFS. Sugar transporter family (TC 2.A.1.1)	2.93	3.70	-
AO090020000694	Belongs to the MFS. Proton-dependent oligopeptide transporter (POT/PTR) family (TC 2.A.17)	3.06	3.29	-
AO090023000039	MFS toxin efflux pump (AflT)	2.93	1.84	-
AO090023000318	C4-dicarboxylate transporter malic acid transport protein	2.18	4.44	2.26
AO090023000411	Ammonium transporter	3.70	-	-
AO090023000430	Ctr copper transporter family	5.25	7.56	-
AO090023000482	MFS transporter	2.21	2.09	-
AO090023000488	Belongs to the MFS. Sugar transporter family (TC 2.A.1.1)	1.87	1.22	-
AO090023000585	MFS multidrug transporter	2.07	3.09	-

Gene ID	Description	Fold change		
		Glc vs. Ace	Glc vs. PAC	Ace vs. PAC
AO090026000101	amino acid transporter	2.48	1.87	-
AO090026000207	MFS transporter	3.38	2.78	-
AO090026000224	Belongs to the MFS	6.10	5.52	-
AO090026000255	K+ potassium transporter	6.17	4.21	-1.96
AO090026000437	mitochondrial phosphate carrier protein	2.82	2.69	-
AO090026000494	Belongs to the MFS. Sugar transporter family (TC 2.A.1.1)	2.73	3.35	-
AO090026000749	Ammonium transporter	1.32	-	-
AO090026000760	monocarboxylate transporter	3.08	2.84	-
AO090026000778	Predicted transporter of the MFS	2.11	2.56	-
AO090038000297	Calcium channel subunit Cch1	1.30	2.02	-
AO090038000314	Ammonium transporter	8.88	10.10	-
AO090038000391	Putative MFS multidrug transporter	1.17	1.06	-
AO090038000535	MFS transporter Fmp42	1.31	1.61	-
AO090038000551	sugar transporter	1.61	2.70	-
AO090038000553	C4-dicarboxylate transporter malic acid transport protein	2.51	-	-1.93
AO090102000073	Monocarboxylate transporter	3.60	-	-
AO090102000135	Putative MFS multidrug transporter	3.22	2.34	-
AO090102000255	Belongs to the MFS. Sugar transporter family (TC 2.A.1.1)	1.99	2.49	-
AO090102000282	Cation efflux protein of the cation diffusion facilitator superfamily	5.98	7.39	-
AO090102000351	Auxin Efflux Carrier superfamily	2.98	2.80	-
AO090102000476	amino acid transporter	1.34	2.15	-
AO090103000075	Belongs to the MFS	4.15	5.45	-
AO090103000099	Belongs to the MFS	1.44	-1.02	-2.45
AO090103000165	Putative MFS siderophore transporter	2.71	7.38	4.67
AO090103000166	ABC multidrug transporter	2.52	4.61	2.10
AO090103000396	Putative MFS multidrug transporter	5.99	8.28	-
AO090120000001	Belongs to the MFS. Proton-dependent oligopeptide transporter (POT/PTR) family (TC 2.A.17)	5.76	7.36	-
AO090120000175	Ctr copper transporter	3.07	3.92	-
AO090120000214	Ctr copper transporter family protein	5.21	6.72	1.50
AO090120000414	Iron-regulated transporter	1.22	1.31	-
AO090120000417	Belongs to the MFS. Sugar transporter family (TC 2.A.1.1)	1.53	1.34	-
AO090120000429	Low affinity iron transport protein	4.07	13.13	9.06
AO090124000012	MFS monocarboxylate transporter	1.79	-	-
AO090124000037	High-affinity iron transporter	3.32	3.82	-

Gene ID	Description	Fold change		
		Glc vs. Ace	Glc vs. PAC	Ace vs. PAC
AO090124000050	Ca^{2+}:H^+ antiporter	4.32	10.42	6.10
AO090124000051	Ca^{2+}:H^+ antiporter	4.29	7.12	2.83
AO090124000052	Ca^{2+}:H^+ antiporter	5.19	11.76	6.57
AO090124000066	MFS transporter	1.40	2.75	1.35
AO090166000026	Amino acid permease	2.69	-	-
AO090701000037	Belongs to the MFS. Sugar transporter family (TC 2.A.1.1)	2.27	1.43	-
AO090701000347	MFS transporter	6.32	5.74	-
AO090701000423	Amino acid transporter	2.38	2.50	-
AO090701000437	MFS domain-containing protein	6.37	5.79	-
AO090701000480	C4-dicarboxylate transporter malic acid transport protein	2.90	2.66	-
AO090701000533	amino acid transporter	1.59	9.12	7.53
AO090701000689	Belongs to the SLC35F solute transporter family	1.12	1.71	-
AO090701000839	Putative transporter of the ABC superfamily	2.15	5.10	2.96
AO090020000267	Permease of the MFS	5.06	4.68	-
AO090001000016	Oligopeptide transporter wykF	-	1.11	-
AO090001000146	Putative MFS transporter	-	6.06	6.58
AO090001000204	MFS domain-containing protein	-	3.87	3.84
AO090001000298	Amino acid transporter	-	1.61	-
AO090001000385	MFS transporter	-	2.09	-
AO090001000510	Belongs to the MIP aquaporin family (TC 1.A.8)	-	2.42	-
AO090003000051	Calcium-transporting ATPase	-	1.11	-
AO090003000167	Putative MFS phospholipid transporter (Git1)	-	1.09	1.21
AO090003000443	H^+/nucleoside cotransporter	-	1.36	-
AO090003000575	Putative MFS monocarboxylate transporter (MCT)	-	2.12	1.61
AO090003000737	MFS transporter	-	5.31	5.96
AO090003000813	MFS monocarboxylate transporter	-	1.63	-
AO090003001081	Cell surface receptor MFS transporter (FLVCR)	-	1.22	-
AO090003001237	Putative transporter of the ABC superfamily	-	2.87	2.03
AO090003001378	Plasma membrane zinc ion transporter	-	1.46	-
AO090003001519	Amino acid permease	-	1.38	-
AO090003001541	Monocarboxylate transporter, MCT family, aspergillic acid transporter	-	5.00	-
AO090005000509	amino acid transporter	-	1.18	1.98
AO090005000693	MFS pantothenate transporter	-	4.33	-
AO090005000769	Belongs to the MFS. Sugar transporter family (TC 2.A.1.1)	-	4.37	-
AO090005000882	ATP-dependent Clp protease	-	1.22	-

Gene ID	Description	Fold change		
		Glc vs. Ace	Glc vs. PAC	Ace vs. PAC
AO090005000968	Belongs to the MFS	-	1.27	1.83
AO090005001027	MFS domain-containing protein	-	2.36	-
AO090005001086	Belongs to the MFS. Sugar transporter family (TC 2.A.1.1)	-	1.66	1.44
AO090005001194	Belongs to the MFS	-	4.57	-
AO090005001495	MFS Transporter	-	4.79	4.95
AO090005001596	Belongs to the MFS	-	1.48	-
AO090009000049	MFS transporter	-	1.83	-
AO090009000061	MFS domain-containing protein	-	1.40	1.19
AO090009000270	MFS transporter	-	1.93	1.58
AO090009000552	Amino acid transporter	-	1.60	1.07
AO090010000101	MFS transporter	-	5.42	-
AO090010000351	amino acid transporter	-2.77	1.26	4.03
AO090010000406	Mitochondrial thiamine pyrophosphate carrier	-	3.03	-
AO090010000470	Belongs to the MFS. Sugar transporter family (TC 2.A.1.1)	-	1.90	-
AO090010000592	Voltage-gated potassium channel	-	2.85	2.11
AO090010000594	Putative MFS transporter	-	4.56	-
AO090010000689	ABC multidrug transporter	-	2.01	-
AO090010000699	Amino acid permease	-	3.61	3.02
AO090011000110	Putative MFS monocarboxylate transporter	-	4.97	-
AO090011000114	Putative multidrug resistance protein, ABC superfamily	-	3.77	3.19
AO090011000116	Amino acid permease	-	3.72	3.03
AO090011000204	Amino acid permease	-	1.27	1.79
AO090011000231	amino acid transporter	-	1.49	-
AO090011000474	MFS transporter	-	3.55	-
AO090011000706	Putative mechanosensitive ion channel	-	2.11	1.14
AO090011000817	Belongs to the NiCoT transporter family (TC 2.A.52)	-	2.56	1.87
AO090011000881	Putative MFS transporter	-	2.17	-
AO090011000912	Magnesium transporter NIPA-domain-containing protein	-	1.03	-
AO090012000048	Belongs to the MFS. Proton-dependent oligopeptide transporter (POT/PTR) family (TC 2.A.17)	-	5.29	-
AO090012000252	MFS transporter	-	1.56	-
AO090012000312	ABC transmembrane type-1 domain-containing protein	-	5.82	-
AO090012000469	Belongs to the MFS	-	1.82	-
AO090012000784	Predicted protein	-	1.29	-
AO090020000354	MFS domain-containing protein	-	2.77	3.27
AO090020000655	Belongs to the MFS. Sugar transporter family (TC 2.A.1.1)	-	2.36	-

Gene ID	Description	Fold change		
		Glc vs. Ace	Glc vs. PAC	Ace vs. PAC
AO090023000092	Belongs to the oligopeptide OPT transporter family.	2.26	-	-
AO090023000988	Belongs to the MFS. Sugar transporter family (TC 2.A.1.1)	1.08	-	
AO090026000005	MFS transporter cpaT	-	6.00	5.34
AO090026000008	Major facilitator superfamily transporter	6.19	5.13	-
AO090026000057	Sugar (and other) transporter	2.36	-	-
AO090026000071	Putative choline transport protein	-	5.48	-
AO090026000078	amino acid transporter	-	8.04	9.37
AO090026000144	MFS domain-containing protein	-	7.36	5.41
AO090026000365	MFS domain-containing protein	-	1.72	-
AO090026000396	MFS multidrug transporter	-	4.54	4.42
AO090026000485	Putative efflux pump, antibiotic resistance protein	-	4.83	-
AO090026000506	MFS domain-containing protein	-	2.01	-
AO090026000775	Belongs to the MFS. Sugar transporter family (TC 2.A.1.1)	-	2.75	-
AO090038000090	Belongs to the MFS. Sugar transporter family (TC 2.A.1.1)	-	2.01	-
AO090038000162	ABC transporter transmembrane region	-	1.84	1.94
AO090038000303	Belongs to the ABC transporter superfamily. ABCG family. PDR subfamily (TC 3.A.1.205)	-	1.55	-
AO090038000490	amino acid transporter	-2.78	3.28	6.05
AO090102000026	MFS monocarboxylate transporter	-	2.36	1.89
AO090102000036	Belongs to the MFS	-	5.62	4.87
AO090102000043	Amino acid permease/ SLC12A domain-containing protein	-	1.54	1.66
AO090102000094	Amino acid permease	-	2.73	-
AO090102000343	Belongs to the TrkH potassium transport family	-	1.86	-
AO090102000401	UNC93-like protein	-	4.39	-
AO090103000310	Belongs to the MFS. Sugar transporter family (TC 2.A.1.1)	-	2.86	-
AO090103000479	Belongs to the oligopeptide OPT transporter family	-	2.76	3.46
AO090103000481	Belongs to the MFS. Sugar transporter family (TC 2.A.1.1)	-	6.16	-
AO090113000038	Major facilitator superfamily transporter	-	2.64	-
AO090113000138	Synaptic vesicle transporter SVOP and related transporters	-	2.05	-
AO090113000139	Belongs to the MFS. Sugar transporter family (TC 2.A.1.1)	-	2.51	-
AO090120000025	MFS domain-containing protein	-	4.98	-
AO090120000093	Magnesium transporter NIPA	-	1.03	-
AO090120000463	Putative MFS monocarboxylic acid transporter	-	1.20	-
AO090138000118	Synaptic vesicle transporter SVOP and related transporters	-	2.23	-

Gene ID	Description	Fold change		
		Glc vs. Ace	Glc vs. PAC	Ace vs. PAC
AO090166000046	oligopeptide transporter	-	3.73	3.46
AO090166000089	MFS sugar transporter	-	2.33	-
AO090701000182	MFS domain-containing protein	-	4.74	4.46
AO090701000190	Putative MFS multidrug transporter	-	1.50	-
AO090701000292	Putative magnesium transporter of the NIPA family	-	1.87	-
AO090701000329	MFS domain-containing protein	-	2.77	-
AO090701000616	ABC transmembrane type-1 domain-containing protein	-	4.24	-
AO090701000617	ABC transmembrane type-1 domain-containing protein	-	5.56	4.92
AO090701000621	Amino acid permease	-	7.67	6.47
AO090701000872	Belongs to the ABC transporter superfamily. ABCG family. PDR subfamily (TC 3.A.1.205)	-	2.54	-
AO090003000798	Sulfate permease	-	-	1.13
AO090005001081	Amino acid permease	-1.70	-	2.21
AO090009000115	Sodium/hydrogen exchanger family-domain-containing protein	-3.58	-	5.03
AO090009000400	UDP-Glc Gal endoplasmic reticulum nucleotide sugar transporter	-	-	1.03
AO090010000743	Belongs to the MFS. Proton-dependent oligopeptide transporter (POT/PTR) family (TC 2.A.17)	-6.91	-2.38	4.53
AO090012000602	ABC fatty acid transporter	-	-	1.52
AO090012000689	MFS transporter	-	-	6.13
AO090012000719	Putative multidrug resistance protein, ABC superfamily	-	-	1.27
