# Supplementary material for: RNA-Seq Based Transcriptome Analysis of Aspergillus oryzae DSM 1863 Grown on Glucose, Acetate and an Aqueous Condensate from the Fast Pyrolysis of Wheat Straw
Source: J Fungi (Basel). 2022 Jul 23;8(8):765. doi: 10.3390/jof8080765 (PMC9394295; doi:10.3390/jof8080765)
Supplement: Supplementary file 1 [file jof-08-00765-s001.zip › Table S2_FC of all genes mentioned in text.pdf]

Table S2: Summary of differentially expressed genes mentioned in this article

Enzyme	EC	Gene ID	Fold change		
			Glc vs. Ace	Glc vs. PAC	Ace vs. PAC
**Amino sugar and nucleotide sugar metabolism**					
glucosamine-phosphate N-acetyltransferase	2.3.1.4	AO090120000132	2.11	1.20	-
glucosamine-6-phosphate deaminase	3.5.99.6	AO090103000020	-1.39	2.06	3.45
chitin synthase	2.4.1.16	AO090026000212	4.56	5.68	-
		AO090026000321	1.42	1.52	-
		AO090026000323	1.36	1.37	-
		AO090005000579	1.27	1.36	-
		AO090011000449	1.26	1.30	-
		AO090012000084	1.56	1.19	-
glutamine-fructose-6-phosphate transaminase	2.6.1.16	AO090003001475	2.88	1.65	-
chitinase	3.2.1.14	AO090102000586	-	3.16	2.17
		AO090003000680	2.13	2.22	-
		AO090103000218	-5.36	-	6.20
α-L-arabinofuranosidase	3.2.1.55	AO090012000298	-	3.93	3.06
UDP-glucose 4-epimerase	5.1.3.2	AO090005001490	-	2.48	2.24
		AO090010000463	-	1.08	-
mannose-6-phosphate isomerase	5.3.1.8	AO090023000719	3.60	4.00	-
**Glycolysis**					
6-phosphofructokinase	2.7.1.11	AO090010000444	1.18	1.26	-
fructose-bisphosphate aldolase	4.1.2.13	AO090009000324	-	-1.57	-
glyceraldehyde 3-phosphate dehydrogenase	1.2.1.12	AO090020000265	6.29	5.66	-
		AO090011000414	2.03	3.46	1.43
enolase	4.2.1.11	AO090003000055	-	1.00	-
pyruvate dehydrogenase E1 component	1.2.4.1	AO090003000290	3.45	3.07	-
α subunit		AO090012000948	-	-1.07	-
dihydrolipoamide dehydrogenase	1.8.1.4	AO090011000486	-	-1.25	-
pyruvate decarboxylase	4.1.1.1	AO090124000047	3.26	2.64	-
		AO090003000661	1.73	1.07	-
aldehyde dehydrogenase (NAD+)	1.2.1.3	AO090009000417	2.55	2.17	-
		AO090026000741	1.32	1.56	-
pyruvate carboxylase	6.4.1.1	AO090023000801	-1.26	-	-
acetyl-CoA synthetase	6.2.1.1	AO090003001112	-	-1.89	-1.63
carnitine O-acetyltransferase	2.3.1.7	AO090001000295	-	-1.32	-1.54
		AO090026000404	-	-1.15	-

**Glyoxylate and TCA cycle**					
citrate synthase	2.3.3.1	AO090102000627	-1.36	-1.87	-
aconitase	4.2.1.3	AO090003000415	-	-1.03	-
isocitrate dehydrogenase (NAD^+) α subunit	1.1.1.41	AO090012000629	-	-1.37	-
isocitrate dehydrogenase (NAD^+) γ subunit		AO090003000008	-	-1.31	-
isocitrate dehydrogenase ($NADP^+$)	1.1.1.42	AO090005001404	-	-1.07	-
α-ketoglutarate dehydrogenase E1 component	1.2.4.2	AO090003001055	-	-1.22	-
α-ketoglutarate dehydrogenase E2 component	2.3.1.61	AO090020000008	-	-1.34	-
		AO090005001174	1.06	1.95	-
succinyl-CoA synthetase β subunit	6.2.1.4<br>6.2.1.5	AO090206000040	-	-1.01	-
succinate dehydrogenase (ubiquinone) flavoprotein subunit	1.3.5.1	AO090020000415	-	-1.10	-
malate dehydrogenase	1.1.1.37	AO090701000013	-1.28	-1.33	-
isocitrate lyase	4.1.3.1	AO090009000219	-2.29	-2.95	-
malate synthase	2.3.3.9	AO090009000557	-1.64	-1.73	-
**Arginine and proline metabolism**					
aspartate aminotransferase	2.6.1.1	AO090120000135	-	-1.36	-
alanine transaminase	2.6.1.2	AO090003000164	-1.39	-1.62	-
glutamate N-acetyltransferase/ amino-acid N-acetyltransferase	2.3.1.35/ 2.3.1.1	AO090701000729	-1.12	-1.39	-
acetylornithine aminotransferase	2.6.1.11	AO090026000394	-	-1.46	-
ornithine carbamoyltransferase	2.1.3.3	AO090023000856	-1.18	-1.98	-
argininosuccinate synthase	6.3.4.5	AO090023000395	-1.59	-1.48	-
argininosuccinate lyase	4.3.2.1	AO090020000418	-1.03	-1.02	-
arginase	3.5.3.1	AO090003000697	1.18	-	-
**Glycine, serine and threonine metabolism**					
betaine-aldehyde dehydrogenase	1.2.1.8	AO090103000021	-	-1.09	-
D-3-phosphoglycerate dehydrogenase/ 2-oxoglutarate reductase	1.1.1.95/ 1.1.1.399	AO090009000711	-1.20	-1.74	-
phosphoserine aminotransferase	2.6.1.52	AO090023000099	-1.88	-1.89	-
phosphoserine phosphatase	3.1.3.3	AO090020000345	-	-1.08	-

**Sulfur metabolism**					
sulfate adenylyltransferase	2.7.7.4	AO090020000349	-1.55	-	-
phosphoadenosine phosphosulfate reductase	1.8.4.8<br>1.8.4.10	AO090020000347	-1.13	-1.39	-
sulfite reductase (NADPH) flavoprotein α-component	1.8.1.2	AO090001000571	-1.62	-1.99	-
sulfite reductase (NADPH) hemoprotein β-component	1.8.1.2	AO090012000271	-1.85	-1.94	-
**Cysteine and methionine metabolism**					
homoserine acetyltransferase/ O-succinyltransferase	2.3.1.3/<br>2.3.1.46	AO090701000235	-1.06	-1.15	-
cystathionine β-synthase	4.2.1.22	AO090011000931	-1.17	-1.35	-
cystathionine γ-lyase	4.4.1.1	AO090103000051	-1.25	-1.63	-
cysteine synthase	2.5.1.47	AO090102000276	-	-1.45	-
5-methyltetrahydropteroyl-triglutamate-homocysteine methyltransferase	2.1.1.14	AO090023000837	-1.33	-	-
**Shikimate pathway**					
3-deoxy-D-arabino-heptulosonic acid 7-phosphate synthase	2.5.1.54	AO090005000086	-1.20	-1.20	-
		AO090005000886	-1.27	-1.21	-
pentafunctional AROM polypeptide	4.2.3.4<br>4.2.1.10<br>1.1.1.25<br>2.7.1.71<br>2.5.1.19	AO090012000502	-	-1.08	-
**Tryptophan synthesis and degradation**					
anthranilate phosphoribosyltransferase	2.4.2.18	AO090003001011	-0.54	-0.95	-
anthranilate synthase	4.1.3.27	AO090012000581	-0.54	-0.93	-
indole-3-glycerol phosphate synthase	4.1.1.48				
phosphoribosylanthranilate isomerase	5.3.1.24				
tryptophan synthase	4.2.1.20	AO090026000284	-1.16	-1.07	-
kynurenine 3-monooxygenase	1.14.13.9	AO090005001567	-1.22	-1.51	-
kynureninase	3.7.1.3	AO090003001247	-1.51	-	-

**Ubiquinone synthesis**					
4-hydroxybenzoate polyprenyltransferase	2.5.1.39	AO090023001001	-	-1.07	-
polyprenyldihydroxybenzoate methyltransferase/ 3-demethylubiquinol 3-O-methyltransferase	2.1.1.114/ 2.1.1.64	AO090003001180	-	-1.12	-
2-methoxy-6-polyprenyl-1,4-benzoquinol methylase	2.1.1.201	AO090001000559	-1.26	-2.03	-
**Terpenoid backbone synthesis and mevalonate pathway**					
hydroxymethylglutaryl-CoA synthase	2.3.3.10	AO090010000487	-	5.60	5.71
hydroxymethylglutaryl-CoA reductase	1.1.1.34	AO090038000218	-3.98	2.48	6.46
		AO090103000311	-	1.38	-
geranylgeranyl diphosphate synthase, type III	2.5.1.1<br>2.5.1.10<br>2.5.1.29	AO090009000093	-	1.95	-
**fatty acid desaturases**					
bifunctional Δ-12/ω-3 fatty acid desaturase	1.14.19.6	AO090010000714	-3.07	-3.12	-
ω-6 fatty acid desaturase / acyl-lipid ω-6 desaturase (Δ-12 desaturase)	1.14.19.6/ 1.14.19.22	AO090001000224	-1.04	-	-
**Pyruvate metabolism**					
D-lactate dehydrogenase (cytochrome)	1.1.2.4	AO090003001006	-	-	-1.75
D-lactate dehydrogenase	1.1.1.28	AO090023000577	-	-	1.11
hydroxyacylglutathione hydrolase (glyoxalase II)	3.1.2.6	AO090120000408	-	-1.07	-1.05
D-lactate dehydratase (glutathione independent glyoxalase)	4.2.1.130	AO090012000129	-	-6.63	-7.49
